# Dynamic Assembly of Microgels and Polymers at Non‐Aqueous Liquid/Liquid Interfaces

**DOI:** 10.1002/advs.202415642

**Published:** 2025-02-07

**Authors:** Xin Guan, Yang Liu, Lianwei Li, Man‐Hin Kwok, Mingming Ding, Hang Jiang, To Ngai

**Affiliations:** ^1^ Department of Chemistry The Chinese University of Hong Kong Shatin, N.T Hong Kong China; ^2^ College of Chemistry and Environmental Engineering Shenzhen University Shenzhen 518060 China; ^3^ College of Polymer Science and Engineering State Key Laboratory of Polymer Materials Engineering Sichuan University Chengdu 610065 China; ^4^ Key Laboratory of Synthetic and Biological Colloids Ministry of Education & School of Chemical and Material Engineering Jiangnan University Wuxi 214122 China

**Keywords:** interfacial assembly, liquid structuring, microgel‐polymer complexes, organogels, Pickering emulsions

## Abstract

Particle assembly at liquid–liquid interfaces presents a promising bottom‐up strategy for creating supramolecular materials with advanced functionalities. However, the significantly lower interfacial tension observed in immiscible organic phases compared to traditional oil–water systems has hindered the effective adsorption and assembly of particles at oil–oil interfaces. In this work, a versatile and effective strategy is presented that utilizes the assembly and jamming of microgels and polymer ligands at non‐aqueous liquid–liquid interfaces to create non‐aqueous Pickering emulsions and reconfigurable droplet networks. The resulting microgel‐polymer complexes form an asymmetric interfacial bilayer with high surface coverage, which effectively minimizes interfacial energy and improves interfacial elasticity. Through a combination of systematic interfacial measurements and molecular dynamics simulations, the underlying mechanisms governing interfacial self‐assembly are elucidated. Notably, the stimuli‐responsive nature of the microgel‐polymer complexes allows for precise control over the interfacial assembly and disassembly by introducing competitive molecules. Furthermore, it is demonstrated that these non‐aqueous Pickering emulsions serve as excellent templates for the fabrication of heterogeneous organogels and microgel‐based colloidosomes through both covalent and non‐covalent crosslinking strategies. This work underscores the potential of non‐aqueous interfaces in advancing materials science and opens new avenues for developing multifunctional materials.

## Graphical abstract

The assembly and jamming of PNIPAM‐*co*‐MAA microgels with NH_2_‐PDMS‐NH_2_ polymer ligands lead to the formation of asymmetric bilayer films at non‐aqueous liquid–liquid interfaces via non‐covalent interactions. The reversible integration of microgels and polymers enables the formatin of reconfigurable droplet networks and stimuli‐responsive non‐aqueous Pickering emulsions, which serve as promising templates for advanced non‐aqueous materials.

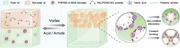

## 1 Introduction

Self‐assembly is a process driven by inter‐ and intramolecular interaction, serving as a versatile bottom‐up strategy for constructing functional materials with superstructures from microscale to macroscale. The complex functions of these assemblies arise from diverse combinations of small building blocks. In biological systems, for instance, proteins with hierarchical structures are formed by the assembly of peptides consisting of amino acids, which play a crucial role in various physiological processes.^[^ ^1^ ^]^ Likewise, the assembly of colloidal particles at liquid–liquid interfaces dominates the properties of droplets, rendering Pickering emulsions as ideal templates for fabricating colloidosomes and other advanced materials.^[^ ^2^ ^]^ However, rigid colloidal particles encounter challenges in spontaneously adsorbing at liquid–liquid interfaces without external energy input due to inherent energy barriers. To promote particle assembly, diverse co‐assembly strategies have been developed, leveraging strong interactions (e.g., electrostatic and host‐guest interactions) between particles and particles, or particles and ligands.^[^ ^3^ ^]^ The reversible non‐covalent interaction between particle assemblies facilitates the design of responsive materials, demonstrating promise in controlled encapsulation and microreactors. Especially, the jamming of polymers or particle surfactants at liquid–liquid interfaces unlocked the possibility of shaping liquids into non‐equilibrium states, allowing the fabrication of complex liquid devices through 3D printing or liquid molding techniques.^[^ ^4^ ^]^ However, an in‐depth understanding of the interaction and the synergistic effect between particles and ligands at oil–oil interfaces is still lacking.

Microgels are defined as soft colloidal particles composed of crosslinked polymer networks. These soft particles can be swollen in good solvents and exhibit characteristics of both rigid particles and surfactants, making them promising stabilizers for Pickering emulsions (sometimes referred to as Mickering emulsions).^[^ ^5^ ^]^ Their inherent softness and deformability improve the coverage at liquid–liquid interfaces, resulting in lower interfacial tension and stronger interfacial rheological properties.^[^ ^6^ ^]^ Upon fine‐tuning the physiochemical properties of microgels, their interfacial assembly patterns can be precisely manipulated, imparting biphasic systems with tailored functionalities.^[^ ^7^ ^]^ Additionally, by utilizing microgel‐stabilized emulsions as templates, microgel‐based colloidosomes (microgelsomes) can be fabricated with stimuli‐responsiveness and semi‐permeability.^[^ ^8^ ^]^ These functionalities allow selective protection and programmed release of specific molecules, shedding light on the design of biomimetic protocells. It is worth noting that, while supramolecular recognition has been developed to facilitate the assembly of anisotropic microgels in aqueous bulk phases, the co‐assembly of microgels and other materials at liquid–liquid interfaces has largely been unexplored.^[^ ^9^ ^]^

In addition to conventional emulsions consisting of oil and water phases, non‐aqueous emulsions have gained increasing attention in special applications where aqueous‐based emulsion systems are incompatible, such as encapsulation of hydrophilic payloads, fabrication of functional materials, and solvent‐free biocatalysis.^[^ ^10^ ^]^ However, unlike oil–water or water–air interfaces, the interfacial tension of oil–oil interfaces was extremely lower, posing significant challenges for the stabilization of non‐aqueous systems.^[^ ^11^ ^]^ Studies have shown that block copolymers and Janus particles with tailored wettability and polarity can be synthesized to stabilize non‐aqueous emulsions.^[^ ^12^ ^]^ More recently, the assembly of cellulose nanocrystal surfactants and molecular brush surfactants has been proposed to structure non‐aqueous liquids.^[^ ^13^ ^]^ While non‐aqueous microgels with specific chemical compositions have been developed, the synthetic pathways to produce these microgels are complex and their effectiveness in stabilizing non‐aqueous Pickering emulsions remains largely unexplored.^[^ ^14^ ^]^ To the best of our knowledge, no attempt has been made to achieve self‐assembly of classical aqueous microgels at oil–oil interfaces, which significantly restricts their potential applications in non‐aqueous biphasic systems, such as structuring liquids and stabilizing emulsions.

Inspired by the assembly of particle surfactants in emulsion formation and liquid structuring, we develop a versatile bottom‐up strategy for the stabilization of non‐aqueous Pickering emulsions through the self‐assembly of complementary carboxyl‐functionalized microgels and diamino‐terminated telechelic polymer ligands. In addition to experimental observations, molecular dynamics (MD) simulations were conducted to model the assembly process and reveal the underlying mechanisms. The asymmetric Janus bilayer was formed by microgel‐polymer complexes at oil–oil interfaces, which was strong enough to structure liquids while retaining soft and semipermeable, thereby enabling the design of reconfigurable droplet networks. The non‐covalent interactions between microgels and polymers allowed stimuli‐responsive disassembly of complexes by introducing competitive ligands, resulting in weakened interfacial films and rapid phase separation. More importantly, the microgel‐stabilized non‐aqueous Pickering emulsions provide a promising platform for architecting heterogeneous organogels. By constructing dynamic hydrogen bonding networks in the continuous phase, non‐covalent organogels can be fabricated with controllable stability and mechanical strength. Furthermore, the interfacial crosslinking of microgel‐polymer complexes allowed the fabrication of covalent organogels with hierarchical structures and non‐aqueous microgelsomes, achieving selective adsorption and permeability to specific molecules.

## 2 Results and Discussion

### 2.1 Synthesis and Physiochemical Properties of Anionic Microgels

Poly(N‐isopropylacrylamide*‐co‐*methacrylic acid) (PNIPAM‐*co*‐MAA) microgels functionalized with carboxyl groups were synthesized via precipitation polymerization (Scheme S1 and Figure S1, Supporting Information). The incorporation of carboxyl groups imparted negative charges to PNIPAM‐*co*‐MAA microgels due to deprotonation (Figure S2, Supporting Information). Notably, it was observed that lyophilized microgels demonstrated excellent dispersibility in dimethylformamide (DMF) solutions, likely attributed to the high affinity of the microgels toward polar solvents. In comparison to various non‐polar and polar solvents, the microgels exhibited the largest diameter in the DMF solution, measuring ≈1.7 µm (Figure S3, Supporting Information). After drying on a silicon wafer, the microgels collapsed and displayed a depressed‐globose shape with a diameter of ≈1 µm and a height of 460 nm (Figure S4, Supporting Information). The swelling volume of PNIPAM‐*co*‐MAA microgels was ≈10 times larger than their dry volume, indicating excellent DMF holding capacity. This swelling behavior suggests that DMF served as a good solvent for PNIPAM‐*co*‐MAA microgels, facilitating microgel dispersion.

### 2.2 Assembly and Jamming of Microgel‐Polymer Complexes at the Oil–Oil Interface

**Figure 1:**
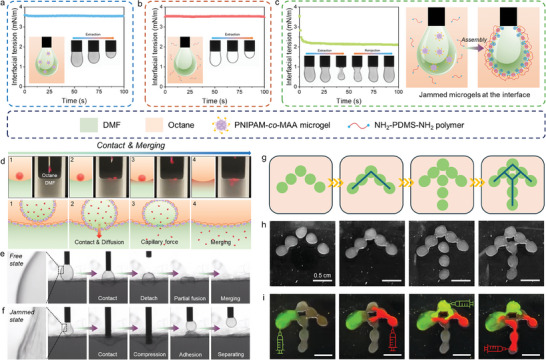
Dynamic IFT of the DMF–octane interface, schematics, and corresponding appearance of pendant drops during deformation in the presence of a) 1 wt.% PNIPAM‐*co*‐MAA microgels in DMF, b) 1 vol% NH_2_‐PDMS‐NH_2_ polymers in octane, and c) 1 wt.% PNIPAM‐*co*‐MAA microgels and 1 vol% NH_2_‐PDMS‐NH_2_ polymers in two immiscible liquids, respectively. d) Schematics and optical images showing the merging process of a dripped droplet contacting the DMF–octane interface covered with microgel‐polymer complexes. e,f) Sequence of snapshots showing the influence of coverage ratio and state of pendant drops on the contact, compression, and separation with the DMF–octane interface covered with microgel‐polymer complexes. g) Schematics and h) optical images of the formation of reconfigurable droplet networks with interpenetrating liquid channels. i) The transportation and communication of dye molecules within the interpenetrating droplet networks. The concentrations of microgels and polymers were 1 wt.% and 1 vol% in two immiscible phases, respectively, for the merging experiments and formation of droplet networks.

To explore the synergistic effect between microgels and polymer ligands, their kinetic adsorption at DMF–octane interfaces was investigated by tracking the dynamic interfacial tension (IFT) using pendant drop tensiometry (Figure S5, Supporting Information). As shown in **Figure** **1**a,b, neither microgels dispersed in DMF nor polymers dissolved in octane were able to reduce the IFT of the DMF–octane interface, indicating no interfacial activity of the sole stabilizer. However, the IFT rapidly reduced from 3.6 to 2.2 mN m^−1^ within a few seconds in the co‐existence of microgels and polymers in the biphasic system. Notably, pronounced wrinkles were observed on droplet surfaces with only a small compression (Figure 1c). Furthermore, these wrinkles did not relax during the process of extraction and reinjection. This result indicates the rapid assembly and jamming of microgel‐polymer complexes at the oil–oil interface, which can be attributed to the high binding energy of carboxyl‐functionalized microgels and diamino‐terminated polymers.

We further investigated the interaction between droplets covered by microgel‐polymer complexes and a flat DMF–octane interface with a thin macroscopic film (Figure S6, Supporting Information). Surprisingly, the droplets stood on the flat interface and maintained their original shape, indicating the strong mechanical strength of the interfacial film. (Figure 1d; Movie S1, Supporting Information). On the contrary, the bare DMF droplet directly merged into the bottom DMF phase upon contact, suggesting the absence of a physical barrier between them (Figure S7, Supporting Information). It is worth noting that dye molecules gradually release out and diffuse into the bottom DMF phase, leading to noticeable color fading in the standing droplet. This phenomenon was analogous to lipid‐based droplet interfacial bilayer (DIB), allowing substance exchange between contacting droplets. Subsequently, the droplet merged with the bottom DMF phase, leading to the complete release of encapsulated dye molecules.

Figure 1e and Movie S2 (Supporting Information) show the process of the contact, compression, and separation of a droplet with a covered DMF–octane interface. Specifically, when the droplet contacted the flat interface, the interfacial film was depressed due to the gravitational effects. A partial fusion was observed at the contact area, resulting in the formation of a liquid bridge between the droplet and the bottom liquid. The connected channel then rapidly expanded due to the capillary forces, ultimately leading to droplet merging (Movie S3, Supporting Information). Based on this phenomenon, we suspect that the coverage ratio and assembly state of microgel‐polymer complexes on droplet surfaces played an important role in the mechanical strength of the interface and the merging behavior. To verify our hypothesis, we simply changed the surface areas by extracting inner liquids from pendant droplets. As a result, the interfacial microgel‐polymer complexes can transition from a free state to a jammed state. As shown in Figure 1f and Movie S4 (Supporting Information), compared to the droplet covered by uniformly distributed microgel‐polymer complexes (coverage≈98%), droplet merging did not occur even under large compression force when the complexes jammed on droplet surfaces (coverage ≈ 100%). It was reported that microgels underwent a phase transition from a non‐close packed structure to a close packed structure during interfacial compression.^[^ ^15^ ^]^ In our case, the interparticle distance of jammed microgel‐polymer complexes was significantly reduced compared to that at a free state, thereby limiting their mobility and rearrangement during droplet contacting.

### 2.3 Reconfigurable Droplet Networks Stabilized by Microgel‐Polymer Complexes

To avoid gravity‐induced droplet merging, individual DMF droplets (10 µL) were distributed in octane solutions. As shown in Figure 1h, a partial fusion occurred when two adjacent droplets were brought into contact. Consequently, interpenetrating dumbbell‐like droplet pairs were obtained without significant shape variation of the original droplets. The controllable fusion between droplets was repeatable, allowing the creation of complex droplet networks with reconfigurability using liquid droplets as building blocks (Figure 1g). It was reported that the coalescence of two Pickering droplets can be arrested when the anisotropic Laplace stress is balanced by the elastic modulus of the jammed interface.^[^ ^16^ ^]^ In our study, once the interconnected liquid channel formed between droplets, microgel‐polymer complexes were able to redistribute and migrate to the open sites on the connected area. As a result, microgel‐polymer complexes transitioned from a free state to a jammed state with decreasing interfacial areas, thereby retaining the integrity of such anisotropic interface and arresting the merging of adjacent droplets.

Additionally, we assume that the transportation of specific substances within the complex droplet networks can be realized owing to its interpenetration. By using fluorescein isothiocyanate (FITC) and Rhodamine B (RB) molecules as the model system, the transportation and mix of different dye molecules within the liquid channel were observed after the injection (Figure 1i; Movie S5, Supporting Information). In addition to the volume increase, the shape of droplet networks remained almost unchanged. While microgels and polymers formed robust assemblies at the interfaces, the semi‐permeable nature of the swollen microgels allowed the exchange of specific molecules across the interface. Specifically, the red color of RB gradually diminished in droplet networks after injection, indicating the transfer of RB molecules between the inner DMF and outer octane phases.

### 2.4 Kinetics and Formation Mechanism of Microgel‐Polymer Interfacial Assemblies

**Figure 2:**
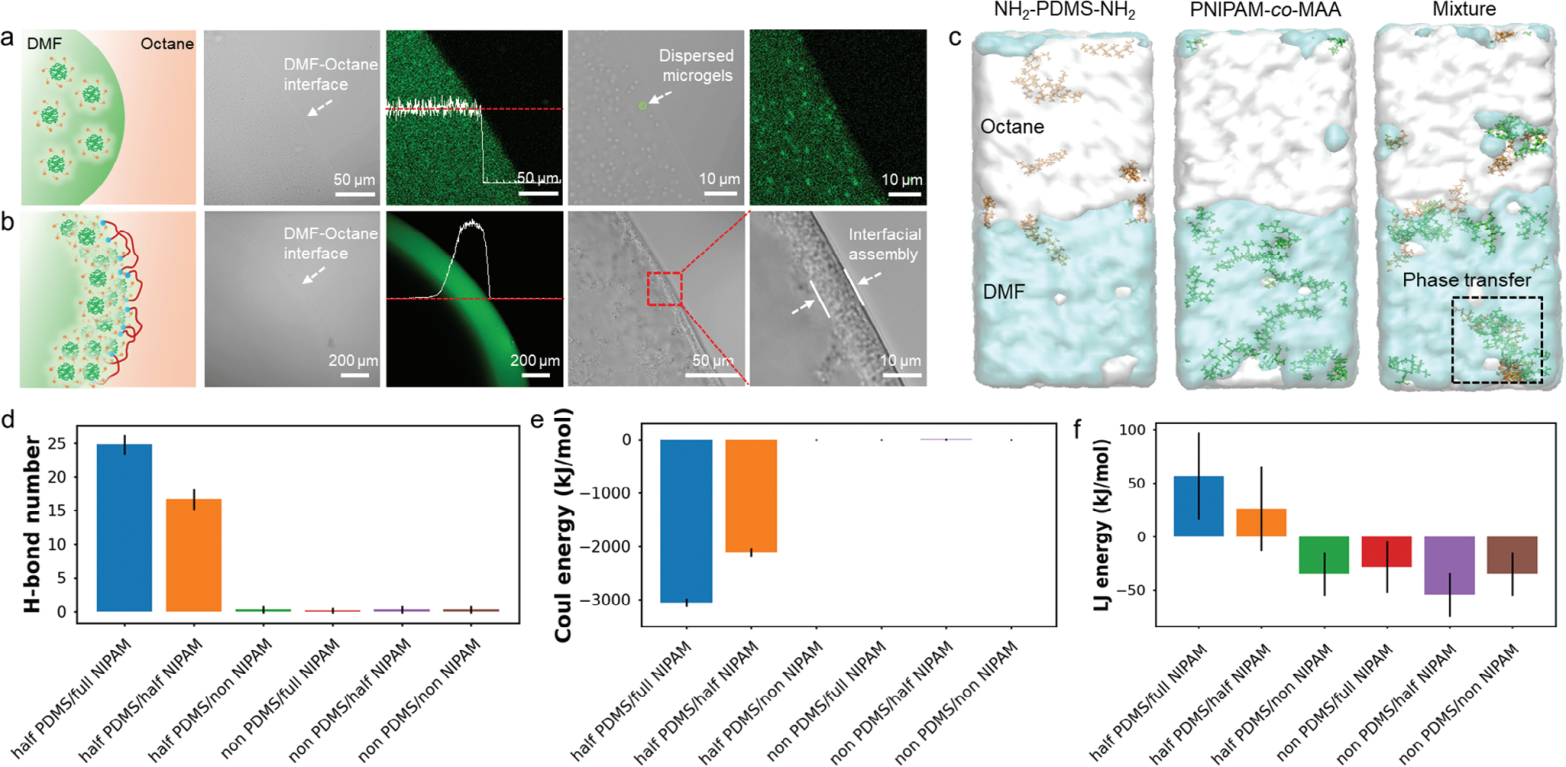
Schematics and CLSM images of a DMF drop containing PNIPAM‐*co*‐MAA microgels surrounded by octane in the a) absence and b) presence of 1 vol% NH_2_‐PDMS‐NH_2_ polymers. The insert plots are the fluorescence intensity of the corresponding CLSM images. c) Last frames of MD simulations for the distribution of PNIPAM‐*co*‐MAA and NH_2_‐PDMS‐NH_2_ molecules near the DMF–octane interface. NH_2_‐PDMS‐NH_2_ was half‐protonated and PNIPAM‐*co*‐MMA was fully deprotonated. Quantification of d) hydrogen bond number, e) Coulomb energy, and f) Van der Waals force between PNIPAM‐*co*‐MMA and NH_2_‐PDMS‐NH_2_ molecules in MD simulations. Different deprotonation and protonation states of PNIPAM‐*co*‐MMA and NH_2_‐PDMS‐NH_2_ molecules were separately considered in different systems for analysis.

The spatial‐temporal distribution and assembly process of microgels in DMF were visualized by CLSM. As shown in **Figure** **2**a, when adding 5 µL DMF dispersion of microgels into the pure octane, FITC‐stained microgels were randomly distributed in the DMF droplet and did not adsorb at the DMF–octane interface. However, if NH_2_‐PDMS‐NH_2_ polymers were introduced in octane, enhanced fluorescence was observed near the interface within a few seconds, indicating the rapid self‐assembly and aggregation of microgels at the interface (Figure 2b). Interestingly, after forming monolayer assemblies at the DMF–octane interface, microgels continued to migrate from the bulk DMF phase to the interface, ultimately resulting in a multilayer structure with an approximate thickness of 10 µm. This phenomenon can be attributed to the strong interactions between oppositely charged microgels and polymer ligands at the interface, as well as the penetration of protonated NH_2_‐PDMS‐NH_2_ polymers into the DMF phase.

To reveal the underlying mechanism of the assembly of microgel‐polymer complexes at the DMF–octane interface, we carried out MD simulations considering the deprotonation and protonation states of the carboxyl and amino groups in PNIPAM‐*co*‐MMA and NH_2_‐PDMS‐NH_2_ molecules, respectively. Specifically, six combinations of the two molecules in different states were investigated. In each simulation, PNIPAM‐*co*‐MMA and NH_2_‐PDMS‐NH_2_ molecules were initially dispersed randomly in the DMF and octane phases, respectively. Following simulations that ran for over 100 ns, we observed that half‐protonated or non‐protonated NH_2_‐PDMS‐NH_2_ molecules predominantly localized in the octane region, while half‐deprotonated or fully deprotonated PNIPAM‐*co*‐MMA molecules were only distributed in the DMF region (Figure 2c). However, when half‐protonated NH_2_‐PDMS‐NH_2_ and fully deprotonated PNIPAM‐*co*‐MMA molecules were simultaneously present in the DMF–octane system, these two types of molecules rapidly assembled at the DMF–octane interface and formed complexes. Additionally, it can be observed that some NH_2_‐PDMS‐NH_2_ molecules bound with PNIPAM‐*co*‐MAA molecules and diffused into the DMF phase, suggesting that interactions between fully deprotonated PNIPAM‐*co*‐MMA and half‐protonated NH_2_‐PDMS‐NH_2_ polymers at the interface can promote the phase transfer process. It should be noted that the lower molecular weight NH_2_‐PDMS‐NH_2_ molecules more easily transferred from the octane phase to the DMF phase in the simulation compared to that with a higher molecular weight (Figure S8, Supporting Information). This phase transfer behavior indicates that low molecular weight NH_2_‐PDMS‐NH_2_ molecules have better solubility in DMF.

To investigate the cross‐interactions between PNIPAM‐*co*‐MMA and NH_2_‐PDMS‐NH_2_ molecules, we calculated the contact numbers among different components in six biphasic systems. As shown in Figure S9 (Supporting Information), the contact number between half‐protonated NH_2_‐PDMS‐NH_2_ and full‐deprotonated PNIPAM‐*co*‐MMA, as well as half‐protonated NH_2_‐PDMS‐NH_2_ and half‐deprotonated PNIPAM‐*co*‐MMA, was notably higher than that between the two molecules in other states. This finding indicates that a certain level of deprotonation and protonation of PNIPAM‐*co*‐MMA and NH_2_‐PDMS‐NH_2_ molecules is crucial for their interfacial assembly. Additionally, we conducted the energy decomposition analysis and assessed hydrogen bond numbers between PNIPAM‐*co*‐MMA and NH_2_‐PDMS‐NH_2_ molecules to elucidate the mechanisms underlying this phenomenon, as illustrated in Figure 2d–f. It was observed that the Coulombic attraction and hydrogen bond numbers were significantly greater in these two combinations compared to the other combinations (Figure 2d,e). When the protonated NH_2_‐PDMS‐NH_2_ molecules attached the DMF–octane interface, the strong electrostatic attraction and hydrogen bonding facilitate the interfacial assembly of deprotonated PNIPAM‐*co*‐MMA molecules, thus anchoring the complexes at the interface. In contrast, while Lennard‐Jones (LJ) energy values for each combination were indeed very similar and close to zero, the distinction between positive and negative values carries important implications (Figure 2f). A positive LJ energy value indicates attraction between two molecules, while a negative value indicates repulsion. Nonetheless, for effective interfacial co‐assembly, it is crucial to note that the Van der Waals forces between these two types of molecules were significantly weaker compared to their electrostatic attractions and hydrogen bonding interactions. This suggests that Van der Waals forces contributed minimally to the assembly phenomenon observed. These simulation results align well with our experimental observations, reinforcing the significance of ionization states and intermolecular interactions in the assembly process of microgel‐polymer complexes at the DMF–octane interface.

### 2.5 Stabilization of Non‐Aqueous Pickering Emulsions

**Figure 3:**
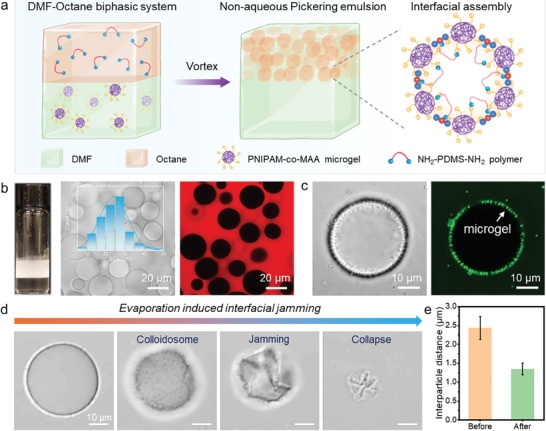
a) Schematics showing the preparation process and stabilization mechanism of non‐aqueous Pickering emulsions. b) Appearance, optical microscopy images, CLSM images, and corresponding droplet size distribution of the octane‐in‐DMF Pickering emulsion co‐stabilized by microgels and polymers. c) Interfacial structure of a droplet laden with microgel‐polymer complexes. d) Solvent evaporation induced morphology evolution of a non‐aqueous Pickering emulsion droplet. e) Interparticle distances of microgels on droplet surfaces before and after solvent evaporation.

We then investigated the emulsification capability of microgels and polymers. Due to the differences in polarity, microgels and polymers were selectively dispersed in DMF and octane phases, respectively (**Figure** **3**a). Before emulsification, RB was incorporated into the DMF solution as a tracer to determine the emulsion type. As shown in Figure 3b, octane‐in‐DMF Pickering emulsions were successfully prepared with an average droplet size of ≈25 µm. Additionally, the droplet surface was covered by FITC‐stained microgel assemblies (Figure 3c). However, the non‐aqueous emulsion cannot be formed in the presence of either microgels or polymers alone. Based on their molecular structures and MD simulation results, we conjecture the stabilization mechanism of emulsions was primarily attributed to the in‐plane electrostatic attraction and hydrogen bonding between microgels and polymers at the interface, which induced the spontaneous formation of asymmetric Janus bilayer films around droplets (Figure 3a).

Surprisingly, it was also observed that the irreversible evaporation of the interior solvent (octane) induced significant interfacial jamming rather than the breakup of non‐aqueous Pickering emulsion droplets, leading to the formation of crumpled droplets with close packed microgel‐polymer complexes (Figure 3d). During solvent evaporation, the interparticle distances between microgels on droplet surfaces significantly decreased due to the reduction of interfacial areas. Specifically, the average interparticle distance between microgels was ≈2.4 µm at the beginning of evaporation, nearly twice the distance measured after evaporation (Figure 3e). This reduction can be attributed to the soft colloidal nature of microgels. Consequently, microgels are deformable and can transition from a non‐close packed arrangement to a close packed hexagonal structure during the surface compression of droplets.^[^ ^17^ ^]^ Furthermore, the corresponding CLSM images indicate that the jamming and buckling of microgels offered structural stability and integrity of the crumpled droplets when dispersing in the DMF solution (Figure S10, Supporting Information).

### 2.6 Responsiveness of Structured Droplets and Non‐Aqueous Pickering Emulsions

**Figure 4:**
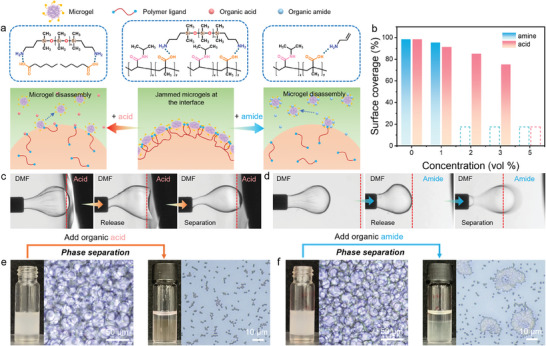
a) Schematics of the assembly and disassembly of microgels and polymers at the oil–oil interface. b) Equilibrium surface coverage of microgel‐polymer complexes in the presence of different concentrations of organic acid and amide in the DMF phase. The influence of c) organic acid and d) organic amine in the morphology evolution of the pendant droplet in DMF solution. Demulsification and phase separation of non‐aqueous Pickering emulsions triggered by the introduction of 5 vol% e) organic acid and f) organic amide.

Molecular recognition plays a crucial role in the self‐assembly and disassembly of host and guest combinations.^[^ ^18^ ^]^ Given the significant influence of electrostatic interactions on the assembly of microgel‐polymer complexes, we hypothesize that the introduction of competitive guest molecules in either protonated or deprotonated states may lead to the formation of new assemblies, thereby resulting in the disassembly of original microgel‐polymer complexes at the DMF–octane interface. To investigate and evaluate the responsiveness of microgel‐polymer complexes, we selected hexanoic acid (HA) and allylamine (AAm) as competitive molecules to represent organic acids and organic amides (**Figure** **4**a). For creating jammed interfacial assemblies, an octane droplet containing polymers was injected into the DMF phase containing microgels (Movie S6, Supporting Information). As shown in Figure 4c and Movie S7 (Supporting Information), when the jammed droplet was manipulated to attach HA molecules at the top of DMF, the interfacial assemblies transitioned from a “solid‐like” to “liquid‐like” state, leading to the wrinkle relaxation and droplet deformation. Subsequently, the octane droplet separated from the tip due to the extremely low interfacial tension and weakened interface. This is because deprotonated HA was able to associate with protonated NH_2_‐PDMS‐NH_2_ polymers at the interface and formed new HA‐polymer complexes, which restricted the assembly of microgels at the interface (Figure 4a). In comparison, a more rapid wrinkle relaxation and droplet separation occurred by introducing AAm molecules in the DMF phase (Figure 4d; Movie S8, Supporting Information). The electrostatic attraction between protonated AAm and deprotonated microgels facilitated their association, leading to the formation of AAm‐microgel complexes both in the DMF phase and at the interface. Additionally, the competitive adsorption of AAm on microgels reduced the anchoring sites and binding energies between microgels and NH_2_‐PDMS‐NH_2_ polymers, resulting in microgel disassembly from the DMF–octane interface (Figure 4a).

Furthermore, the effects of HA and AAm on the surface coverage of microgel‐polymer complexes were investigated. Figure 4b indicates the threshold concentrations of HA and AAm for efficient and stable formation of microgel‐polymer complexes on droplet surfaces. In the presence of 1 vol% AAm, the interaction between microgels and polymers was sufficiently strong to stabilize the DMF–octane interface (Figure S11a, Supporting Information). Consequently, the surface coverage only underwent a slight reduction. In comparison, it seems that HA had less impact on the formation of microgel‐polymer complexes. Microgel‐polymer complexes were able to form at 3 vol% HA, whereas the disassembly and wrinkle relaxation occurred immediately due to the enhanced in‐plane compression and weaker binding energies (Figure S11b, Supporting Information). Upon increasing the concentration of AAm and HA above 2 vol% and 5 vol%, respectively, no wrinkles were observed during extraction, indicating that microgel‐polymer complexes did not form at the interface.

The addition of organic acid and amide also significantly affected the stability of as‐prepared non‐aqueous Pickering emulsions. Specifically, complete phase separation occurred within a few seconds after introducing 5 vol% HA or AAm into the emulsion system (Figure 4e,f). This rapid phase separation was attributed to the disassembly of microgel‐polymer complexes at droplet surfaces (Movies S9 and S10, Supporting Information). Following HA‐induced demulsification, the disassembled microgels were observed to be randomly distributed in the DMF phase (Figure 4e). However, AAm‐induced demulsification caused aggregation of microgels in the DMF phase, which was likely due to the enhanced interparticle attraction and the weakened electrostatic repulsion due to the association of AAm molecules in the microgel matrix (Figure 4f).

### 2.7 Influencing Factors for Non‐Aqueous Pickering Emulsion Formation

We further investigated the factors influencing the formation of non‐aqueous Pickering emulsions by adjusting different emulsification parameters, such as the stabilizers, DMF–octane ratio, and oil compositions. As shown in Figure S12 (Supporting Information), NH_2_‐PDMS‐NH_2_ polymers with molecule weights ranging from 1 to 27K were utilized to prepare emulsions. Notably, only those polymers with an intermediate molecular weight (Mw 3K) were effective in stabilizing octane‐in‐DMF Pickering emulsions with PNIPAM*‐co‐*MAA microgels. We then explored the possibility of preparing DMF‐in‐octane Pickering emulsions at lower DMF/octane volume ratios. Unexpectedly, at DMF/octane volume ratios of 1/2 or 1/3, stable emulsions cannot be obtained in all systems (Figure S13, Supporting Information). We observed that NH_2_‐PDMS‐NH_2_ polymers with a high molecular weight (Mw 27K) were able to invert the interfacial curvature, thus facilitating the formation of an unstable DMF‐in‐octane Pickering emulsion in the presence of PNIPAM*‐co‐*MAA microgels (Figure S14, Supporting Information). However, despite microgels remaining adsorbed at the interface, droplet coalescence occurred rapidly after emulsion preparation and eventually led to phase separation (Figure S15, Supporting Information). Based on our observations from MD simulations and a previous study,^[^ ^3d^ ^]^ we suspect that low molecular weight NH_2_‐PDMS‐NH_2_ polymers (Mw 1K) were more likely to transfer from the interface to the polar oil phase (DMF), rather than form a stable and robust assemblies with microgels at the interface. This phase transition behavior can be primarily attributed to the lower interfacial desorption energy, higher level of protonation, as well as the electrostatic attraction exerted by deprotonated PNIPAM*‐co‐*MAA microgels. In contrast, NH_2_‐PDMS‐NH_2_ polymers with a high molecular weight (Mw 27K) exhibited a significantly greater affinity toward the non‐polar oil phase (octane). Consequently, their adsorption and protonation at the DMF–octane interface were restricted, which in turn limited their ability to associate with PNIPAM*‐co‐*MAA microgels for stabilizing the interface.

The formation of non‐aqueous Pickering emulsions can also be achieved by adjusting the oil compositions (Table S1, Supporting Information). The creaming, sedimentation, and transmittance of as‐prepared Pickering emulsions can be finely regulated using the oil phases with specific polarity, density, and reflective index (Figure S16, Supporting Information). We also explored the versatility of this strategy to prepare non‐aqueous Pickering using different types of particles. The results indicate that the architecture of PNIPAM*‐co‐*MAA microgels did not affect their assembly and association with NH_2_‐PDMS‐NH_2_ polymers at the interface (Figure S17a, Supporting Information). However, no emulsions can be formed when using amino‐functionalized microgels, hydrophilic silica nanoparticles, and carboxylate polystyrene particles as the stabilizers. This finding suggests that intermolecular electrostatic attraction and particle softness are crucial factors in the formation of interfacial particle‐polymer complexes and the stabilization of non‐aqueous Pickering emulsions (Figure S17b, Supporting Information).

### 2.8 Fabrication of Non‐Covalent POs

**Figure 5:**
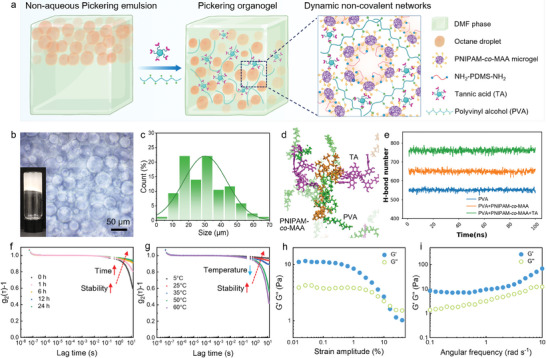
a) Schematics of the construction of non‐aqueous POs and the hydrogen bonding dominated dynamic non‐covalent networks. b) Appearance and optical microscopy image of an octane‐in‐DMF PO. c) Size distribution of droplets in octane‐in‐DMF POs. d) Snapshot of PVA/PNIPAM‐*co*‐MAA/TA system in MD simulations. Hydrogen bonds are represented by black dot lines. e) The variation of hydrogen bond number in the system containing different molecules. Normalized intensity autocorrelation function versus lag time for an octane‐in‐DMF PO f) over time and g) at different temperatures. h) Strain amplitude sweep and i) angular frequency sweep for oscillatory shear complex moduli (G' and G'') for an octane‐in‐DMF PO at room temperature.

Emulsion gels exhibit the characterizations of both emulsions and polymer‐based gels, making them useful across various applications in food, biomedicines, and functional materials.^[^ ^19^ ^]^ An emulsion gel can be formed by either covalent or non‐covalent intermolecular interactions. Among various strategies, hydrogen bonding allows the construction of dynamic non‐covalent gel networks within the aqueous or organic solutions. However, Pickering emulsion‐based organogels (POs) have been rarely reported due to the challenges in stabilizing non‐aqueous Pickering emulsions. In this study, we utilized microgel‐polymer complex‐stabilized octane‐in‐DMF Pickering emulsions as templates to prepare non‐covalent POs by introducing polyvinyl alcohol (PVA) polymers and tannic acid (TA) in the DMF phase (**Figure** **5**a). Unlike the octane‐in‐DMF Pickering emulsions, which experienced inevitable creaming after preparation (Figure 1b), the as‐prepared POs exhibited enhanced stability and maintained a constant appearance during storage (Figure 5b; Figure S18, Supporting Information). This stability can be attributed to the significantly increased viscosity after gelation, which effectively trapped the emulsion droplets within the gel networks, thereby limiting their movement. However, the droplets in POs became larger with a wider size distribution (Figure 5c). Based on the molecular structures of various components in the POs (Figure S19, Supporting Information), we conjecture that the formation of gel networks primarily resulted from multiple hydrogen bonds between the carboxyl groups in PNIPAM*‐co‐*MAA microgels and the hydroxyl groups in PVA and TA molecules. Additionally, given that the octane droplets were covered by a monolayer of PNIPAM*‐co‐*MAA microgels (Figure 1c), hydrogen bonding also existed between the dispersed droplets and the continuous gel matrix.

MD simulations were conducted to verify our conjecture. By randomly solvating PVA, PVA/PNIPAM‐*co*‐MAA, and PVA/PNIPAM‐*co*‐MAA/TA molecules in DMF, aggregation of these molecules was observed in all three systems (Figure 5d). Notably, the addition of PNIPAM‐*co*‐MAA and TA molecules efficiently increased the number of hydrogen bonds in the PVA system (Figure 5e). Specifically, the PVA/PNIPAM‐*co*‐MAA/TA system had ≈100 more hydrogen bonds than the PVA/PNIPAM‐*co*‐MAA system, despite the addition of only 6 TA molecules. This simulation result suggests that TA molecules contributed a substantial number of hydrogen bonds, highlighting their critical role in organogel formation.

Based on dynamic non‐covalent hydrogen bonding and polymer chain entanglement, the reconfigurable gel networks also allowed the mediation of organogel stability and mechanical strength. The storage and thermal stability of non‐covalent POs were evaluated using diffusing‐wave spectroscopy. Figure 5f indicates that the droplets were still moveable in the freshly prepared POs at room temperature, while their movement was significantly restricted after 6 h of storage. Additionally, the stability of POs was enhanced at lower temperatures (Figure 5g). Specifically, the intensity autocorrelation function remained almost unchanged at 5 °C even at a long lag time. In contrast, the rupture of hydrogen bonds led to a faster decay of the autocorrelation function at the temperature above 50 °C due to the enhanced movability of droplets. Unlike conventional microgel‐stabilized oil‐in‐water Pickering emulsions, the non‐aqueous Pickering emulsion droplets maintained stability at high temperatures without coalescence or phase separation (Figure S20, Supporting Information). This observation confirms that high temperatures did not induce the desorption of microgel‐polymer complexes from the interface.

The mechanical properties of the non‐covalent POs were further assessed through oscillatory shear measurements. The POs performed either linear or non‐linear viscoelastic behaviors under different strain amplitudes. The elastic modulus (G′) was greater than the viscous modulus (G″) within the range of low strain amplitudes (<10%), indicating that the mechanical properties of the POs were predominantly governed by the elasticity (Figure 5h). Additionally, both G′ and G″ remained relatively constant in the linear regime while undergoing significant reduction near the crossover strain point (G′ = G″). Moreover, G' was larger than G″ within the range of frequency from 0.1 to 10 rad/s, with no crossover between G' and G″ (Figure 5i). This result indicates that the non‐covalent POs exhibited solid‐like viscoelasticity, with droplets being tightly entrapped inside the gel matrix. The POs also demonstrated a typical shear‐thinning behavior with an increase in angular frequency (Figure S21, Supporting Information). The reduction of complex viscosity at a higher angular frequency can be attributed to the more significant polymer chain relaxation and droplet deformation in the continuous phase. Consistent with the temperature effect on stability, POs exhibited higher elasticity at a low temperature, likely due to the strengthened hydrogen bonding interactions (Figure S22a, Supporting Information). Additionally, the higher microgel concentration, an increased internal phase fraction, and the use of highly viscous oil all contributed to enhanced elasticity and viscosity of the resulting POs (Figure S22b–d, Supporting Information). Therefore, these strategies offer effective means for the tunable control of the mechanical strength of non‐covalent POs.

### 2.9 Fabrication of Covalent POs and Non‐Aqueous Microgelsomes

**Figure 6:**
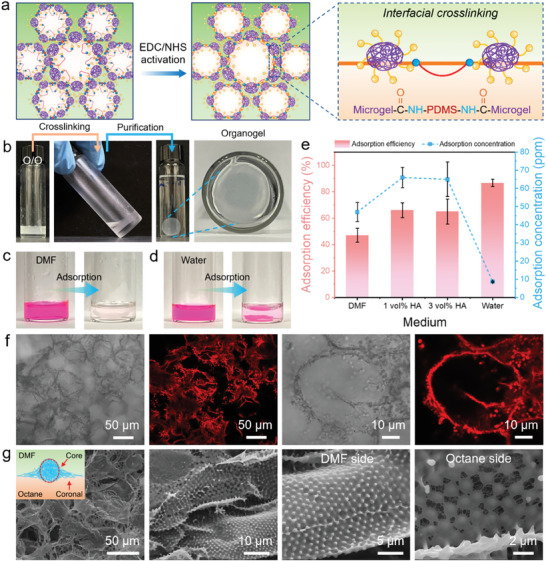
a) Schematics of the interfacial crosslinking of microgels and polymers on droplet surfaces for the fabrication of microgelsomes and covalent POs. b) Optical images showing the morphology, crosslinking, and purification of covalent POs. The feasibility of using covalent POs as adsorbents for the removal of organic dye molecules (RB) in c) DMF and d) water environment. e) Adsorption efficiency and concentration of RB in different media. f) Optical microscopy and CLSM images of covalent POs assembled by RB‐dyed microgels and polymers. g) SEM images showing the differences in microstructures of covalent POs on the opposite sides of the DMF–octane interfaces.

In addition to the development of non‐covalent POs, we assumed that covalent POs can be fabricated through interfacial crosslinking rather than polymerization of monomers in the continuous phase. As illustrated in **Figure** **6**a, carbodiimides (EDC) were dissolved in DMF to activate carboxyl groups in PNIPAM*‐co‐*MAA microgels for direct conjugation with primary amines in NH_2_‐PDMS‐NH_2_ polymers at oil–oil interfaces, leading to the formation of amide bonds. The introduction of N‐hydroxysuccinimide (NHS) improved the crosslinking efficiency by generating stable amine‐reactive intermediates during the process.

Initially, we attempted to fabricate covalent POs using non‐covalent POs as templates. However, despite maintaining unchanged morphology, the gel structure of covalent POs was unstable, leading to collapse during the purification with ethanol (Figure S23, Supporting Information). This instability can be attributed to isolated droplets in the non‐covalent gel networks. As a result, only the interfaces of connected droplets can be crosslinked while the interconnected covalent networks cannot be constructed in the continuous phase. To overcome this challenge, we employed creaming non‐aqueous Pickering emulsions as an alternative template. The enlarged contact areas between droplets facilitated the formation of covalent gel networks assembled by microgels and polymers, serving as the organogel skeleton (Figure 6b). The morphology and interfacial microstructure of covalent POs were further visualized by CLSM and SEM. Figure 6f shows that droplets were tightly bound together with densely packed microgels on the surface. Moreover, analogous to microgel adsorbing at oil–water interfaces, microgels at the DMF–octane interface maintained a well‐defined core‐corona architecture with a more pronounced protrusion in the polar DMF phase, indicating a greater affinity of microgels for the polar DMF phase compared to the nonpolar octane phase (Figure 6g).

Furthermore, the covalent POs can be transferred to the aqueous phase and turned into Pickering emulsion‐based hydrogels (PHs). In comparison to ethanol‐swollen POs, the size and liquid holding capability of water‐swollen PHs underwent significant reductions (Figure S24, Supporting Information). Leveraging the hierarchical porous structure and hydrogen bonding interactions, RB molecules can be removed in both DMF and aqueous solutions (Figure 6c,d). It was observed that the absorbance of RB molecules varied significantly in different media, which may be related to the polarity of the medium and the ionization state of RB molecules (Figure S25, Supporting Information). Additionally, the presence of a low concentration of HA was found to enhance the adsorption concentration and efficiency of RB by covalent POs (Figure 6e). This increase is likely due to the strengthened hydrogen bonding between protonated carboxylic groups in both PNIPAM*‐co‐*MAA microgels and RB molecules. However, the adsorption concentration of RB in the aqueous phase was significantly lower than that in DMF, indicating a better removal efficiency of RB by covalent POs in organic solutions. Beyond the fabrication of covalent POs, non‐aqueous microgelsomes were templated from non‐aqueous Pickering emulsion droplets with semi‐permeability, allowing for the diffusion of non‐polar perylene molecules (Figure S26a, Supporting Information). The CLSM images of microgelsomes indicate that microgels were fixed on the surfaces as a monolayer after interfacial crosslinking, providing stability to microgelsomes in different environments (Figure S26b, Supporting Information).

## 3 Conclusion

In summary, we demonstrate the assembly and jamming of microgel‐polymer complexes at non‐aqueous liquid–liquid interfaces. The formation of elastic and asymmetric bilayer interfacial film is facilitated by intermolecular non‐covalent attractions between deprotonated PNIPAM*‐co‐*MAA microgels and protonated NH_2_‐PDMS‐NH_2_ polymers. The strong binding energy between complementary microgels and polymers enables the stabilization of stimuli‐responsive non‐aqueous Pickering emulsions and the creation of reconfigurable droplet networks. Consequently, the controllable assembly and disassembly of microgel‐polymer complexes at the oil–oil interface can be achieved by utilizing protonated and deprotonated ligands, leading to rapid phase separation. Moreover, by employing non‐aqueous Pickering emulsions as templates, heterogeneous organogels, and microgel‐based colloidosomes can be successfully fabricated via either covalent or non‐covalent crosslinking methods. In comparison to conventional non‐aqueous emulsions stabilized by tailored Janus particles or block copolymers, our study offers an alternative and efficient strategy for stabilizing non‐aqueous biphasic systems, which opens new avenues for the design and fabrication of stimuli‐responsive non‐aqueous microreactors and organogels.

## Conflict of Interest

The authors declare no conflict of interest.

## Author Contributions

X.G. and Y.L. contributed equally to this work. X.G. performed conceptualization, methodology, investigation, formal analysis, and wrote the original draft. Y.L. performed simulation, formal analysis, wrote the original draft. L.W.L. performed review and editing. M‐H.K. performed review and editing. M.M.D. performed review and editing. H.J. performed supervision, acquired funding acquisition, reviewed and edited the final manuscript. T.N. performed conceptualization, supervision, project administration, acquired funding acquisition, reviewed and edited the final manuscript. All authors discussed the results and commented on the manuscript.

## Supporting information



Supporting Information

Supplemental Movie 1

Supplemental Movie 2

Supplemental Movie 3

Supplemental Movie 4

Supplemental Movie 5

Supplemental Movie 6

Supplemental Movie 7

Supplemental Movie 8

Supplemental Movie 9

Supplemental Movie 10

## Data Availability

The data that support the findings of this study are available from the corresponding author upon reasonable request.
