# Supplementary material for: Dynamic Assembly of Microgels and Polymers at Non‐Aqueous Liquid/Liquid Interfaces
Source: Adv Sci (Weinh). 2025 Feb 7;12(13):2415642. doi: 10.1002/advs.202415642 (PMC11967781; doi:10.1002/advs.202415642)
Supplement: Supplementary file 1 — Supporting Information [file ADVS-12-2415642-s004.docx]

Supporting Information

Dynamic Assembly of Microgels and Polymers at Non-Aqueous Liquid/Liquid Interfaces

Xin Guan, Yang Liu, Lianwei Li, Man-Hin Kwok*, Mingming Ding, Hang Jiang* and To Ngai*

Experimental Procedures

Materials

*N*-Isopropylacrylamide (NIPAM, 99%), poly(vinyl alcohol) (PVA, 88% hydrolyzed), tannic acid (TA, 95%), and fluorescein isothiocyanate (FITC, 90%) were bought from J&K Scientific. *N, Nʹ*-Methylenebisacrylamide (MBA, 99%), Rhodamine B (RB, 99%), perylene (99%), and octane (99%) were from Acros Organics. Potassium persulfate (KPS) was from Dieckmann Chemical Industry Co., Ltd. Bis(3-aminopropyl) terminated poly(dimethylsiloxane) (NH_2_-PDMS-NH_2_, M_w_ 1K, 3K, and 27K) was brought from Shanghai Macklin Biochemical Technology Co., Ltd. Dimethylformamide (DMF, 99.8%) was obtained from RCI Labscan Ltd. Methacrylic acid (MAA), N-(3-dimethylaminopropyl)-Nʹ-ethylcarbodiimide hydrochloride (EDC, 98%) and squalene (98%) was received from TCI Co., Ltd. Hexadecane (98%), N-hydroxysuccinimide (NHS) was purchased from Shanghai Aladdin Bio-Chem. Triethylamine (TEA, 99%) was obtained from Scharlau. Ethanol (≥ 99.9%), dodecane (99%), squalane (98%), fluoresceinamine, isomer I was from Sigma-Aldrich. All chemicals were directly used without further purification. Deionized water (Milli-Q grade, 18.2 MΩ cm) was used in this work.

Synthesis and purification of fluorescent PNIPAM-*co*-MAA microgels

The synthetic procedure for anionic PNIPAM*-co-*MAA microgels was performed as follows: Initially, 1.2 g of the monomer (NIPAM), 0.032 g of the crosslinker (MBA), and 150 μL of the comonomer (MAA) were dissolved in 60 mL of deionized water and then transferred to a 125 mL three-neck bottle flask. The reaction solution was placed in a water bath and heated to 70 °C with purging nitrogen gas for 30 mins. Following this, 1 mL of a KPS initiator solution (5 wt%) was injected to the reaction solution to initiate free radical precipitation polymerization. Once the formation of precursor microgels started, the appearance of the solution turned from transparent to translucent. The synthetic procedure would be completed after 5h. After cooling the reaction solution to room temperature (25 °C), the microgel dispersion was collected. Unreacted monomers and low molecular weight oligomers were removed by high-speed centrifugation at 12000 rpm and the centrifugated microgels were redispersed in deionized water. The centrifugation and redispersion process would be repeated 4 times for thorough purification of microgels.

To impart green fluorescence to PNIPAM-*co*-MAA microgels. 20 mL of the non-stained microgels dispersion (0.5 wt%) was activated with 30 mg of NHS and 40 mg of EDC at pH 5 for 20 mins. Subsequently, TEA was added to adjust the pH of the solution to approximately 7.4, followed by adding 0.5 mg of fluoresceinamine to react with the carboxylic groups in microgels. The reaction would proceed overnight to obtain the fluorescent PNIPAM-*co*-MAA microgels. The fluorescent microgels were purified by aforementioned centrifugation and redispersion process at least 4 times.

Construction of non-aqueous Pickering emulsions

For the construction of non-aqueous Pickering emulsions, immiscible DMF and octane were selected as the two model oil phases. Before emulsification, 0.25 wt% PNIPAM*-co-*MAA microgels and 1 wt% NH_2_-PDMS-NH_2_ polymers were dispersed in DMF and octane, respectively. Then, DMF dispersion was mixed with octane at 2,700 rpm for 10 s to get the octane-in-DMF Pickering emulsion. To determine the continuous phase of the emulsion, Rhodamine B, a fluorescent dye, was used to stain the DMF phase in the non-aqueous Pickering emulsion.

Fabrication of non-covalent POs

The fabrication of non-covalent POs was achieved through the establishment of dynamic hydrogen bonding networks in the continuous phase. Initially, 0.5 wt% lyophilized PNIPAM*-co-*MAA microgels were dispersed in 950 μL DMF solution to form microgel dispersion. Subsequently, 8 wt% PVA (88% hydrolyzed) was dissolved in the DMF solution by heating at 70 °C for 6 hours. Then, the DMF solution containing microgels and PVA was mixed with octane containing 1 wt% NH_2_-PDMS-NH_2_ at 2700 rpm for 10s (DMF/octane volume ratio = 1/2). To induce gelation, 50 μL DMF solution containing 10 wt% TA was introduced in the non-aqueous biphasic system, resulting in the formation of the non-aqueous Pickering emulsion exhibiting gel feature.

Fabrication of covalent POs and microgelsomes

The fabrication of covalent POs and microgelsomes was accomplished through interfacial crosslinking of microgel-polymer complexes. Following the formation of non-aqueous Pickering emulsions, a certain amount of EDC and NHS was added to the emulsion system to initiate the crosslinking reaction. The molar ratio of EDC, NHS, and carboxylic groups in microgels was maintained as 2:2:1. In terms of covalent POs, the emulsion droplets underwent creaming and aggregation to ensure interfacial connections among the droplets. In contrast, covalent microgelsomes were fabricated by gentle shaking of the emulsion to prevent droplet contact, thereby allowing for the formation of stable capsulated structures.

Measurement of dynamic interfacial tension and surface coverage

An all-purpose contact angle measuring & contour analysis system (OCA 25, Dataphysics) equipped with corresponding SCA 25 software was utilized to record and measure the dynamic interfacial tension using the “pendant drop” method at room temperature (25 °C). Specifically, a 10 µL drop of DMF was suspended in the octane solution for continuous measurement. The Young-Laplace equation was applied to analyze the captured drop shape, thus enabling the calculation of dynamic interfacial tension.

To determine the surface coverage of microgel-polymer complexes, a pendant droplet was created by injecting a 10 µL DMF solution containing 1 wt% PNIPAM-*co*-MAA microgels into the octane solution containing 1 wt% NH_2_-PDMS-NH_2_ polymers. After reaching the assembly equilibration, the microgel-polymer complexes were uniformly distributed on droplet surfaces (termed the free state). With the reduction of the pendant droplet surface areas through extraction, the microgel-polymer complexes became crowded and jammed at the interface, leading to wrinkle formation. The coverage (*C*) on the droplet surface in the free state could be estimated as *C ≈ S_J_/S_F_*, where *S_J_* and *S_F_* are surface areas of the jammed and free states respectively, which can be directly measured using the tensiometes.

Characterizations of microgels and emulsion

Dynamic light scattering (DLS, Malvern ZS90) was used to measure the size distribution and zeta potential of PNIPAM*-co-*MAA microgels in various medis at 25 °C. Fourier-transform infrared spectroscopy (FTIR) was used to analyze the chemical composition of PNIPAM*-co-*MAA microgels. An optical microscope (OLYMPUS, Japan) was used to capture the morphology of as-prepared emulsions and microgels on the substrate. The confocal micrographs of emulsions were taken with a Nikon Eclipse Ti inverted microscope (Nikon, Japan). FITC and Rhodamine B were excited by lasers at wavelengths of 488 and 543 nm, respectively. The morphology of microgels was visualized using a Quanta 400F (FEI Company) scanning electron microscope (SEM) equipped with a field emission electron gun at 10 kV. The surface features and height profiles of microgels and microgelsomes in their dry state were measured by an atomic force microscope (AFM, Digital Instruments NanoScope IVa) in tapping mode, using a cantilever with 50-70 kHz resonance frequency and 1-5 N/m spring constant.

Stability and rheological characterizations of non-covalent POs

The stability of non-covalent POs with varying compositions was evaluated by diffusing wave spectroscopy (DWS Rheolab, Switzerland). Measurements were conducted over a range of storage times and temperatures to assess the stability of different samples. Bulk rheological measurements were conducted on a Malvern Kinexus Lap + rheometer equipped with parallel plates of 20 mm diameter. For the oscillatory shear measurement, the parameters of strain amplitude, frequency, and gap size were set as 0.1 %, 1 s^−1^, and 0.5 mm, respectively.

Removal of dye molecules using covalent POs

The purified covalent POs were freeze-dried before usage. Typically, lyophilized POs were added to either a DMF solution or an aqueous solution containing RB molecules at varying concentrations. The concentration of fluorescent dye in solutions and corresponding adsorption efficiency were determined by measuring the highest absorbance at specific wavelengths using a UV−visible spectrometer (Alpha-1, Shanghai Lab-Spectrum Instruments Co., Ltd.), both before and after the removal process.

Molecular dynamics simulations

We carried out all the simulations using GROMACS 2021 software^[1]^ and the CHARMM Generalized Force Field (CGenFF)^[2]^. The time step employed in the simulation was 2 fs. The Lennard-Jones interactions were smoothly switched off between 10 and 12 Å by a forced-based switching function. Long-range electrostatic interactions were calculated using the particle-mesh Ewald (PME) method^[3]^ with a direct space tolerance of ${10}^{-5}$. Langevin Integrator was applied, and the temperature was maintained at 298K. For the investigation of the distributions of PNIPAM-*co*-MMA and NH_2_-PDMS-NH_2_ molecules with different protonation states in the biphasic systems of octane and DMF, the Berendsen barostat was used to maintain the semi-isotropic simulation cells at 1 bar in the z-axis, while the compressibility in the x and y-axes was set to zero. For the investigation of the aggregation mechanism of PVA, PNIPAM-*co*-MMA and TA molecules in DMF, the Berendsen barostat was used to maintain the isotropic simulation cells at 1 bar in all directions. The force field parameters used in this study were generated using the CHARMM-GUI website server^[4]^ and the starting structure was built with the Packmol package^[5]^.

In each simulation of PNIPAM-*co*-MMA and NH_2_-PDMS-NH_2_ molecules distribution, a total of 1200 DMF molecules and 600 octane molecules were introduced into a simulation box with dimensions of 5 nm×5 nm×13 nm. After the phase separation of DMF and octane, 10 PNIPAM-*co*-MMA molecules, 10 NH_2_-PDMS-NH_2_ molecules, and a mixture of 10 PNIPAM-*co*-MMA/10 NH_2_-PDMS-NH_2_ molecules were randomly added to their respective systems. In the half-protonated NH_2_-PDMS-NH_2_ systems, one of the amino groups connected to NH_2_-PDMS-NH_2_ molecules was protonated. Additionally, in the half and fully deprotonated PNIPAM-*co*-MAA systems, either half or all of the carboxyl groups in MMA were deprotonated. For comparison, 60 PVA molecules, 60 PVA/18 PNIPAM-*co*-MMA molecules and 60 PVA/18 PNIPAM-*co*-MMA/6 TA molecules were introduced into a larger simulation box containing 7500 DMF molecules with dimensions of 10 nm×10 nm×10 nm for investigating the interactions between PVA, PNIPAM-*co*-MAA and TA molecules.

Statistical Analysis

All values are expressed as the mean ± the standard error of the mean (s.e.m). Data was analyzed using Origin 2022 software.

Results and Discussion

**
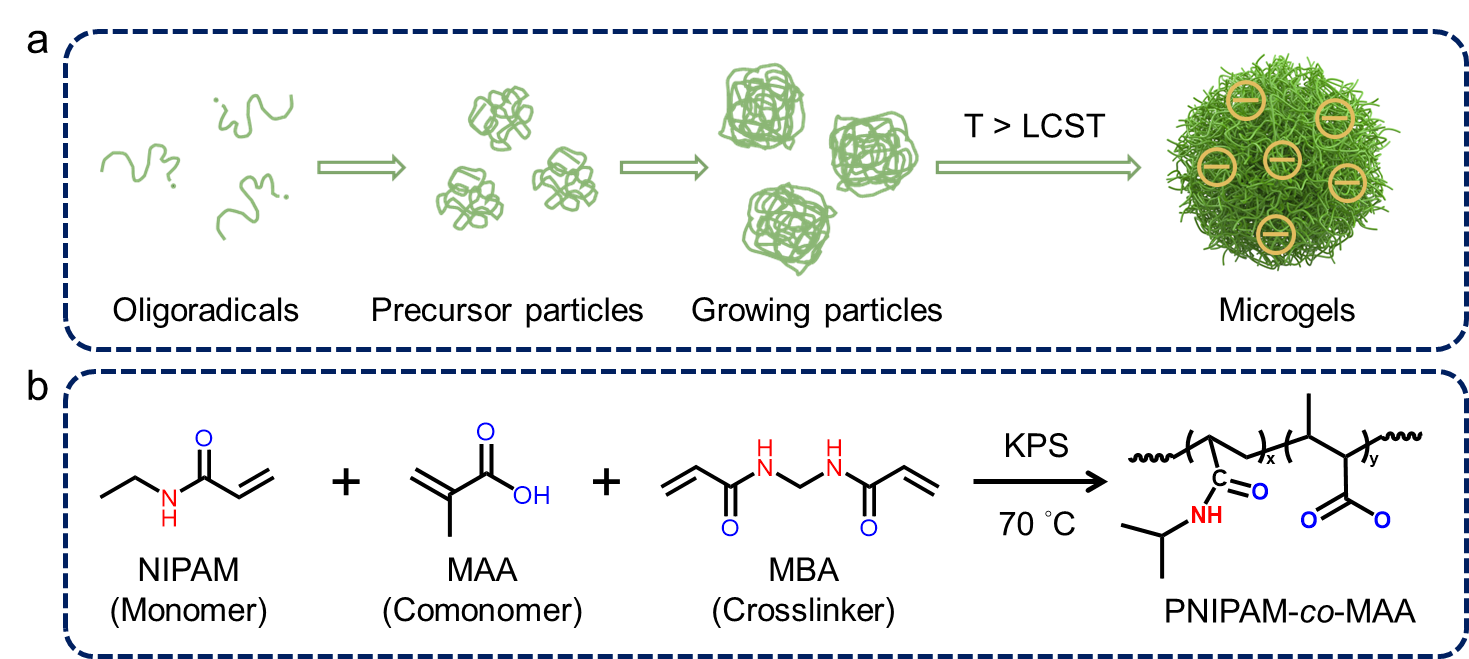
**

Scheme S1. (a) Typical synthetic route of microgels via precipitation polymerization. (b) Reagents and reaction conditions of PNIPAM*-co-*MAA microgel synthesis.

Table S1. Oil phases investigated in this work to demonstrate the feasibility of non-aqueous Pickering emulsion formation.

|  | *Ethanol* | *DMF* | *Octane*  *(C_8_)* | *Dodecane*  *(C_12_)* | *Hexadecane (C_16_)* | *Squalane*  *(C_30_)* |
| --- | --- | --- | --- | --- | --- | --- |
| *Polarity* | Polar | Polar | Non-polar | Non-polar | Non-polar | Non-polar |
| *Density (g/cm^3^)* | 0.789 | 0.944 | 0.703 | 0.750 | 0.773 | 0.810 |
| *Refractive index* | 1.362 | 1.431 | 1.398 | 1.422 | 1.434 | 1.452 |

**
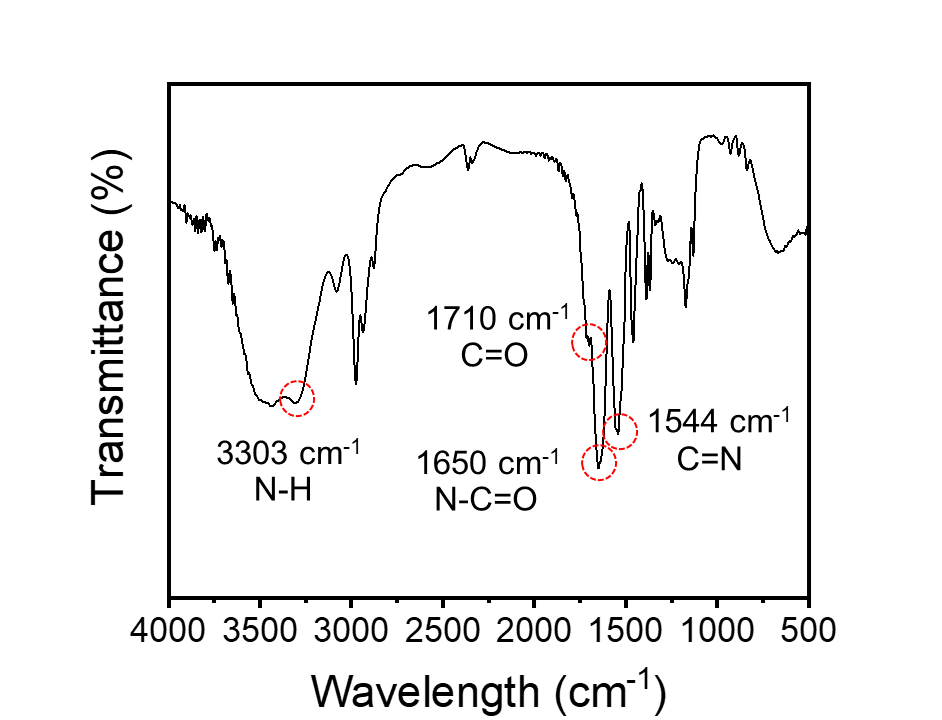
**

Figure S1. FTIR spectra of PNIPAM*-co-*MAA microgels within the scanning range of 500–4000 cm^–1^.

Figure S2. Zeta potential of PNIPAM*-co-*MAA microgels in an aqueous solution.


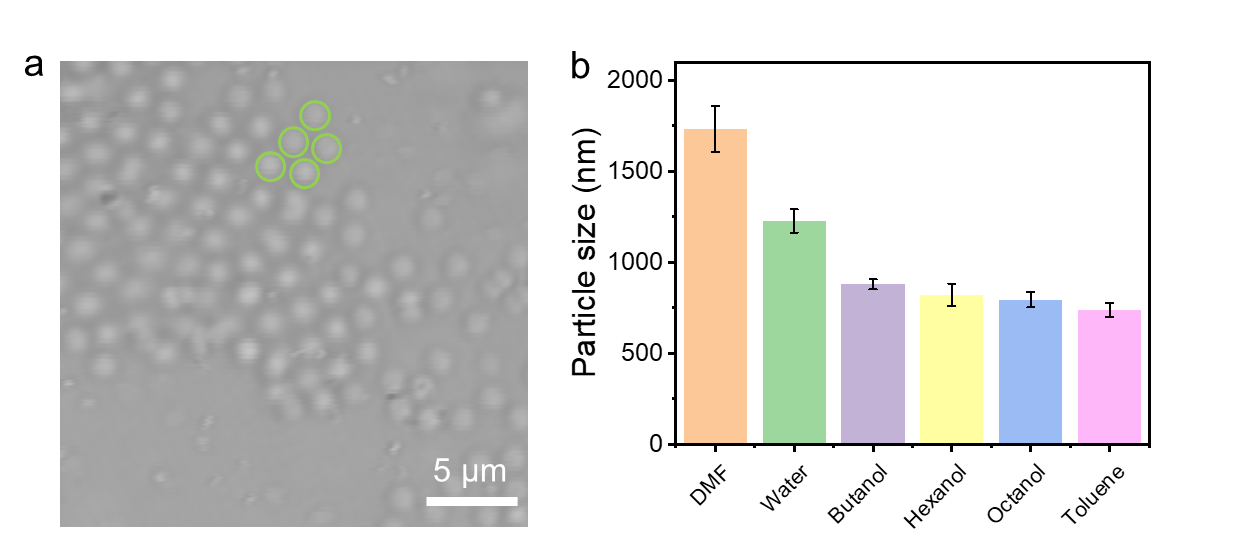


Figure S3. (a) Optical microscopy image of PNIPAM*-co-*MAA microgels dispersing in a DMF solution. (b) Average particle size of swollen PNIPAM*-co-*MAA microgels in different media.


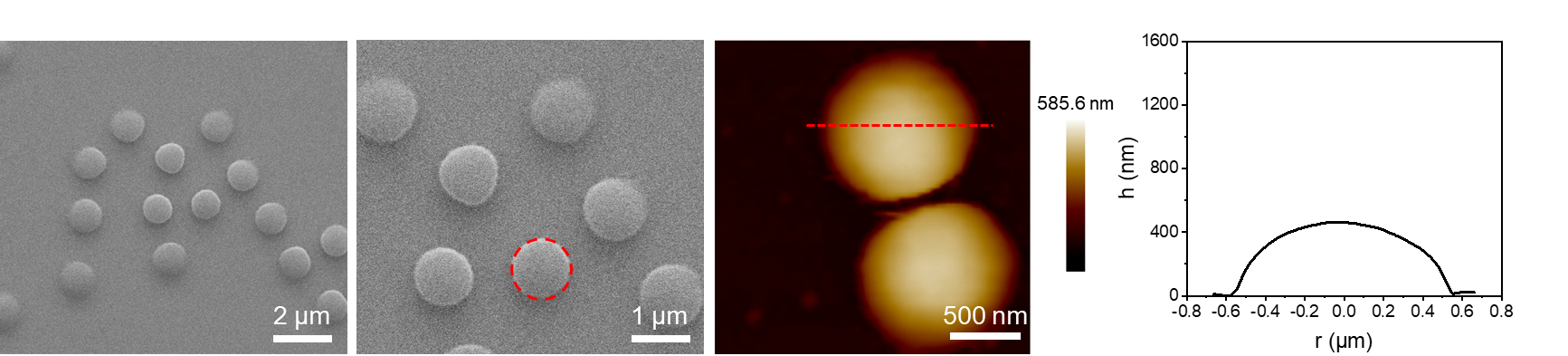


Figure S4. SEM images, AFM image, and the corresponding height profile of PNIPAM*-co-*MAA microgels on a silicon wafer after drying at room temperature.


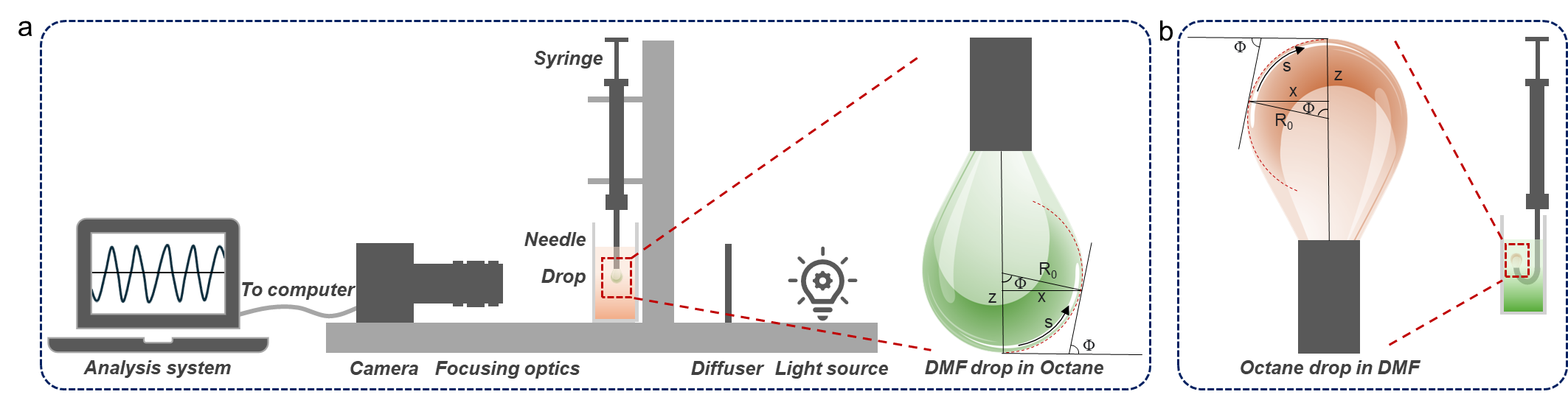


Figure S5. The setup of pendant drop tensiometer for dynamic interfacial tension measurement and observation for droplet morphology evolution. (a) A DMF drop suspended in the octane phase. (b) An octane drop suspended in the DMF phase.


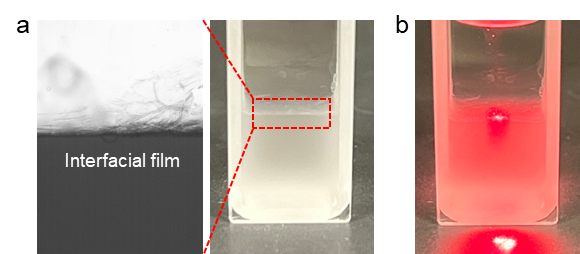


Figure S6. (a) Optical image of 2D macroscopic film consisting of microgel-polymer complexes at a flat DMF-octane interface. (b) Laser irradiation on the interfacial macroscopic film.


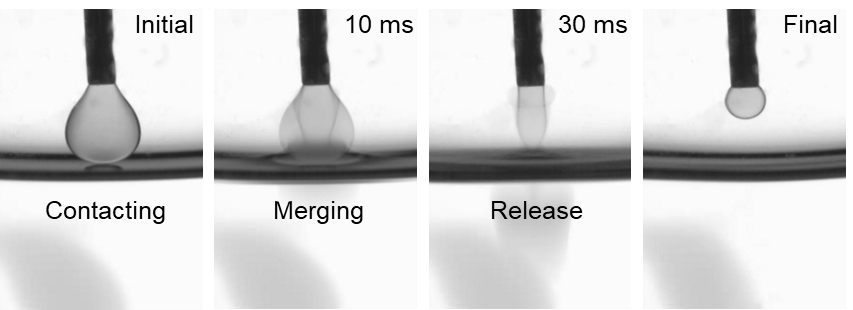


Figure S7. Sequence of snapshots showing the process of droplet merging in the absence of microgel-polymer complexes at the interface.


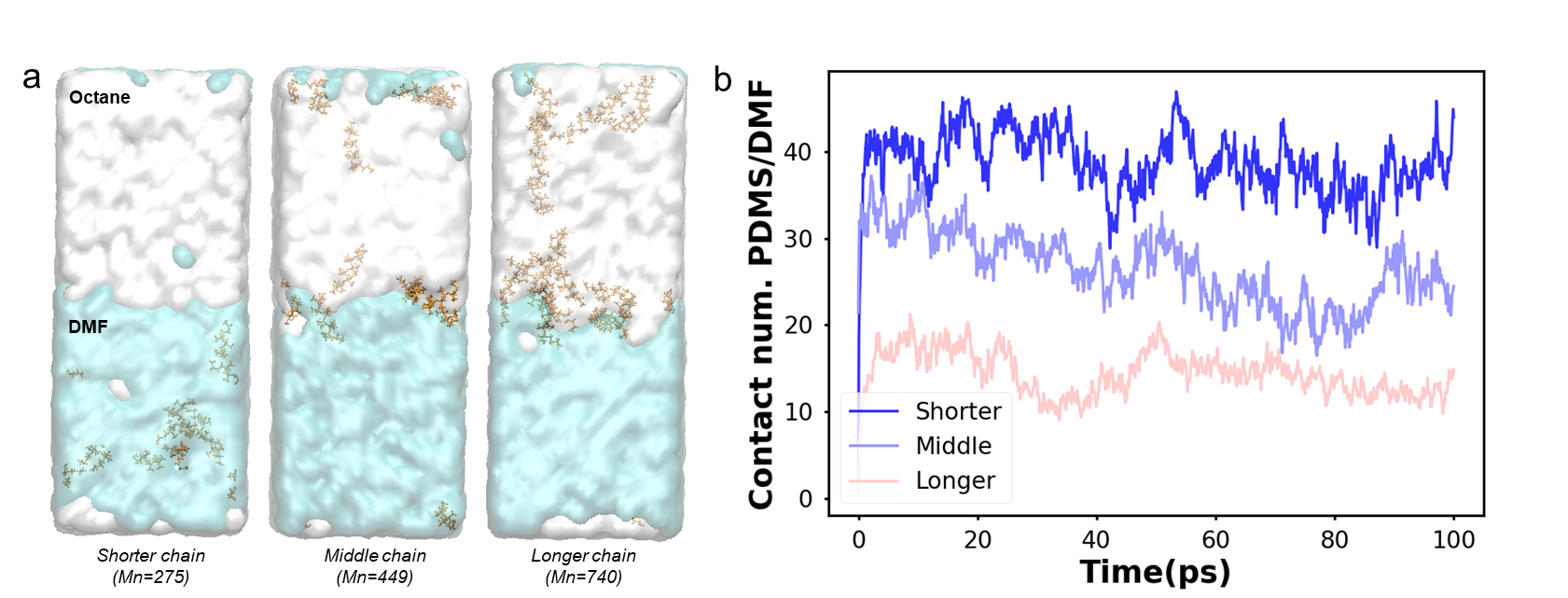


Figure S8. (a) Last frames of MD simulations for the distribution of NH_2_-PDMS-NH_2_ molecules with different molecular weight in the DMF–octane system. (b) Contact number between NH_2_-PDMS-NH_2_ molecules and DMF phase in MD simulations. NH_2_-PDMS-NH_2_ was half-protonated and initially dispersed in the octane phase.


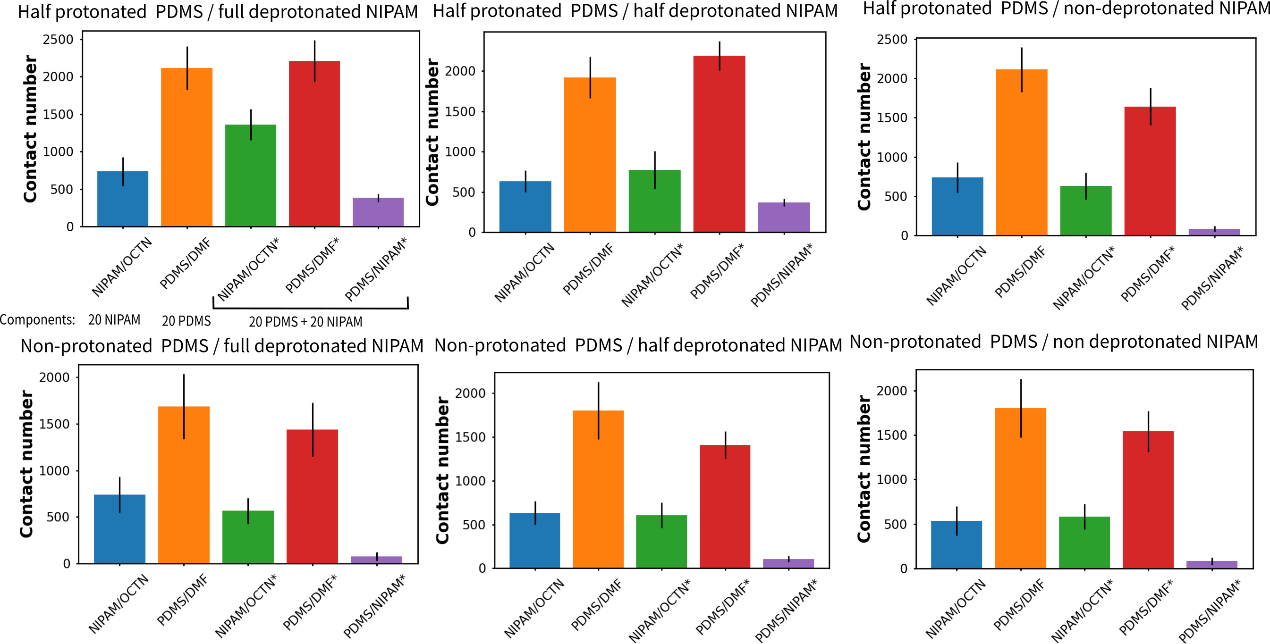


Figure S9. Contact number of different systems in MD simulation. Different protonation states of PNIPAM*-co-*MMA and NH_2_-PDMS-NH_2_ molecules were investigated. The first to fifth collum represent contact numbers between different components.


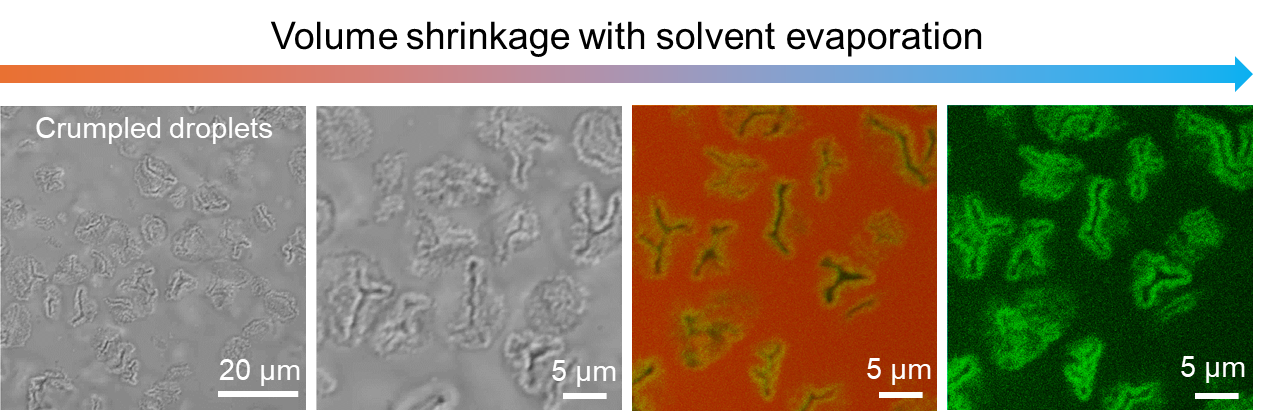


Figure S10. Optical microscopy images and CLSM images of crumpled droplets stabilized by microgel-polymer complexes in a DMF solution.


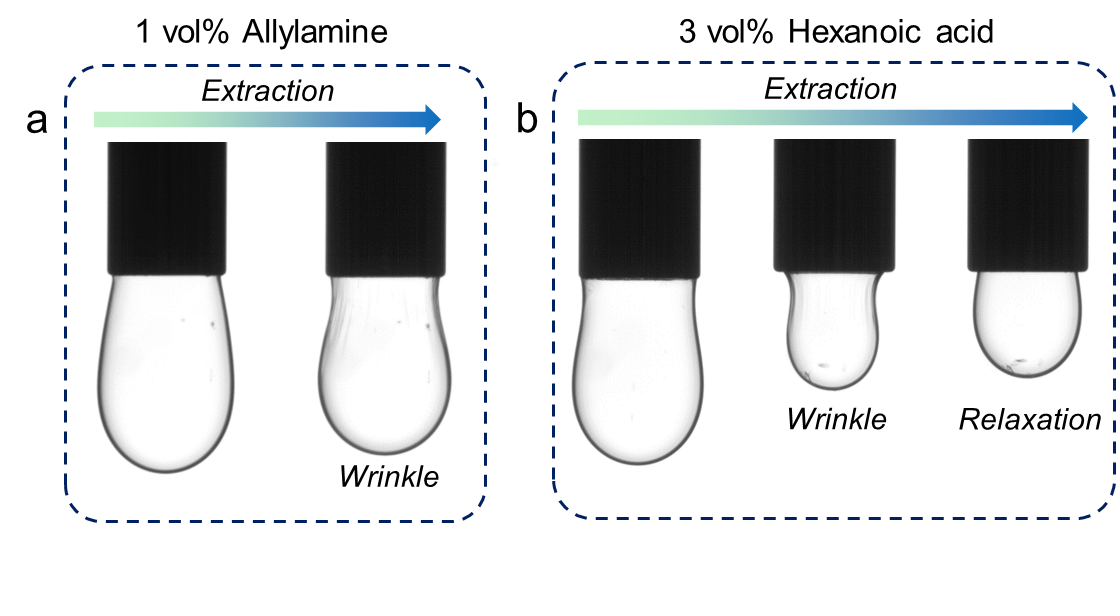


Figure S11. Sequence of snapshots showing the process of droplet compression during volume extraction. (a) 1 vol% AAm and (b) 3 vol% HA were introduced into the DMF phase, respectively.


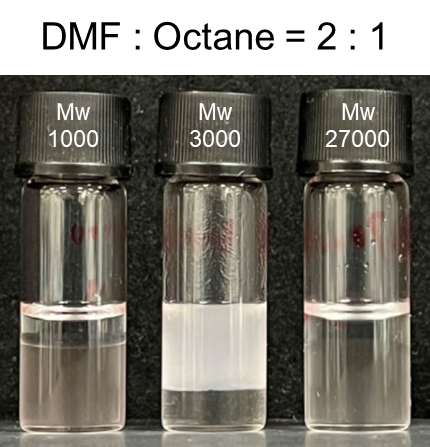


Figure S12. Optical images of emulsified DMF-octane biphasic systems containing 0.25 wt% PNIPAM*-co-*MAA microgels and 1 wt% NH_2_-PDMS-NH_2_ polymers with different molecule weights ranging from 1K to 27K. The DMF/octane ratio was maintained at 2/1.


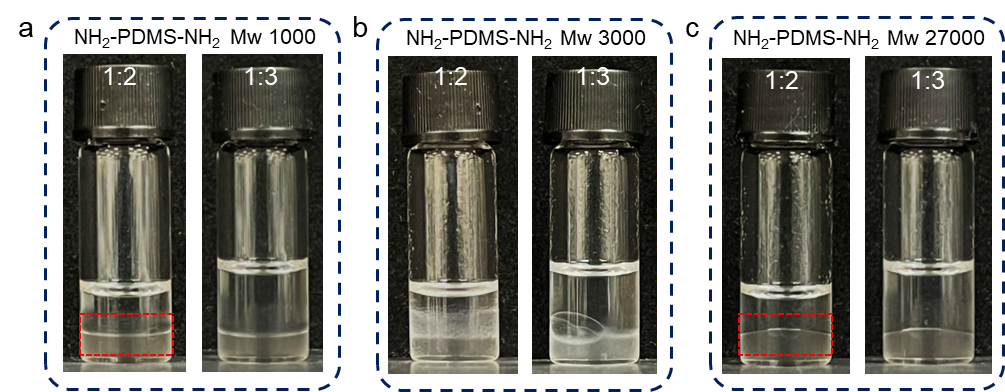


Figure S13. Optical images of emulsified DMF-octane biphasic systems with different DMF/octane ratios ranging from 1/2 to 1/3. The biphasic systems contained 0.25 wt% PNIPAM*-co-*MAA microgels and 1 wt% NH_2_-PDMS-NH_2_ polymers with (a) Mw 1K, (b) Mw 3K, and (c) Mw 27K.


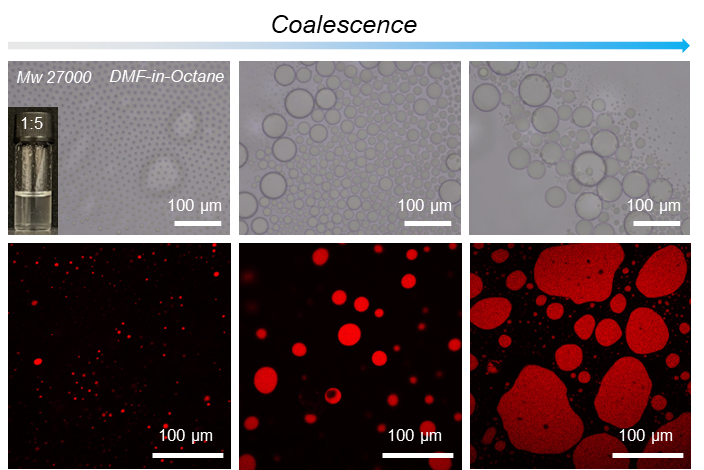


Figure S14. The morphology evolution of a DMF-in-octane Pickering emulsion containing 0.25 wt% PNIPAM*-co-*MAA microgels and 1 wt% NH_2_-PDMS-NH_2_ polymers (Mw 27K) with an increasing storage time. The DMF/octane ratio was maintained at 1/5.


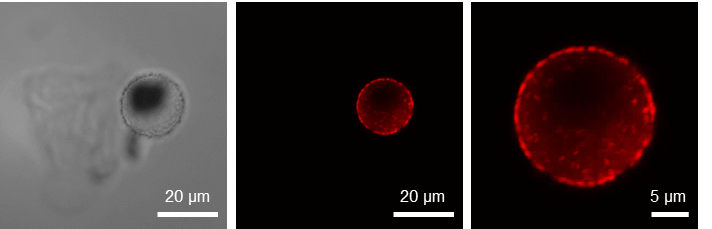


Figure S15. Optical microscopy image and corresponding CLSM images of a DMF-in-octane Pickering emulsion droplet stabilized by PNIPAM*-co-*MAA microgels and NH_2_-PDMS-NH_2_ polymers (Mw 27K).


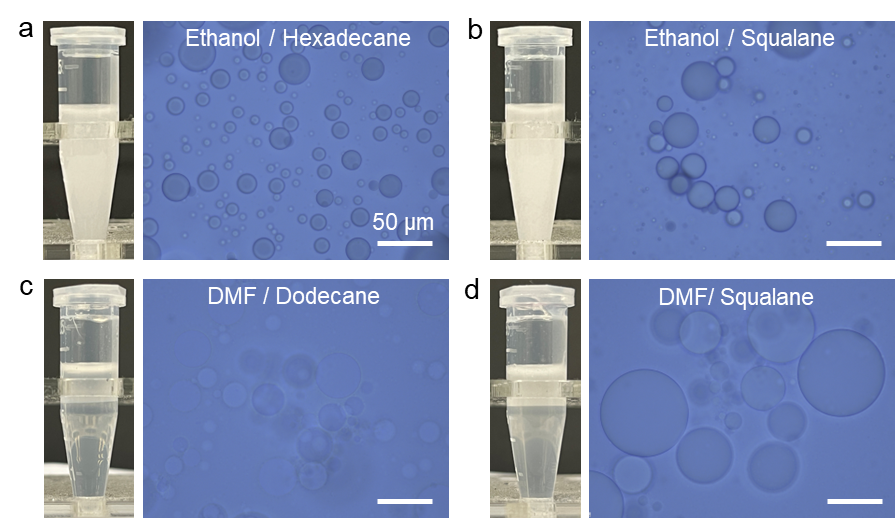


Figure S16. Appearance and optical microscopy images of non-aqueous Pickering emulsions stabilized by 0.25 wt% PNIPAM*-co-*MAA microgels and 1 wt% NH_2_-PDMS-NH_2_ polymers (Mw 3K). The emulsion contained different combinations of polar and non-polar oil phases, including (a) ethanol and hexadecane, (b) ethanol and squalane, (c) DMF and dodecane, and (d) DMF and squalane.


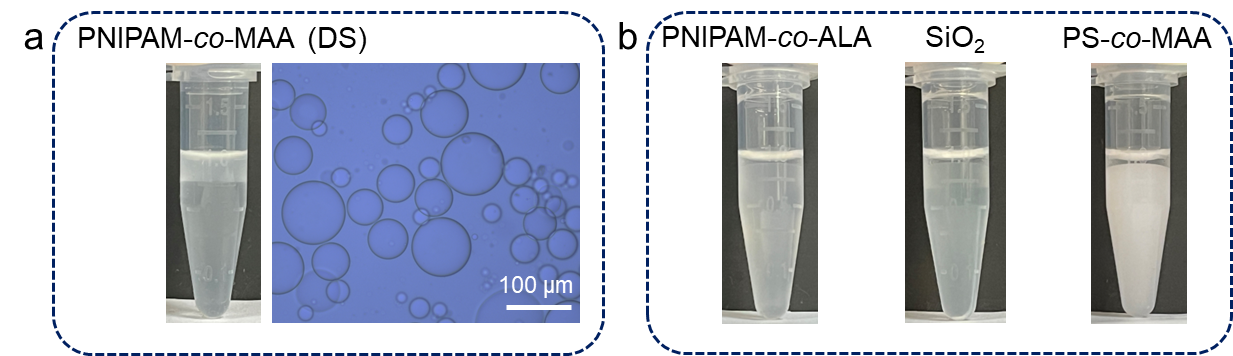


Figure S17. (a) Appearance and optical microscopy image of an octane-in-DMF Pickering emulsion stabilized by 0.25 wt% DS microgels and 1 wt% NH_2_-PDMS-NH_2_ polymers (Mw 3K). (b) Appearance of the emulsified DMF-octane biphasic systems containing 0.25 wt% different particles and 1 wt% NH_2_-PDMS-NH_2_ polymers.


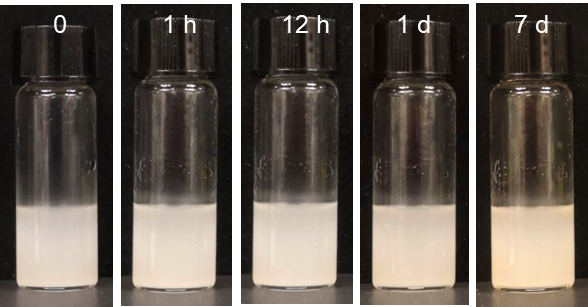


Figure S18. Appearance of a non-covalent octane-in-DMF PO after storage for different times


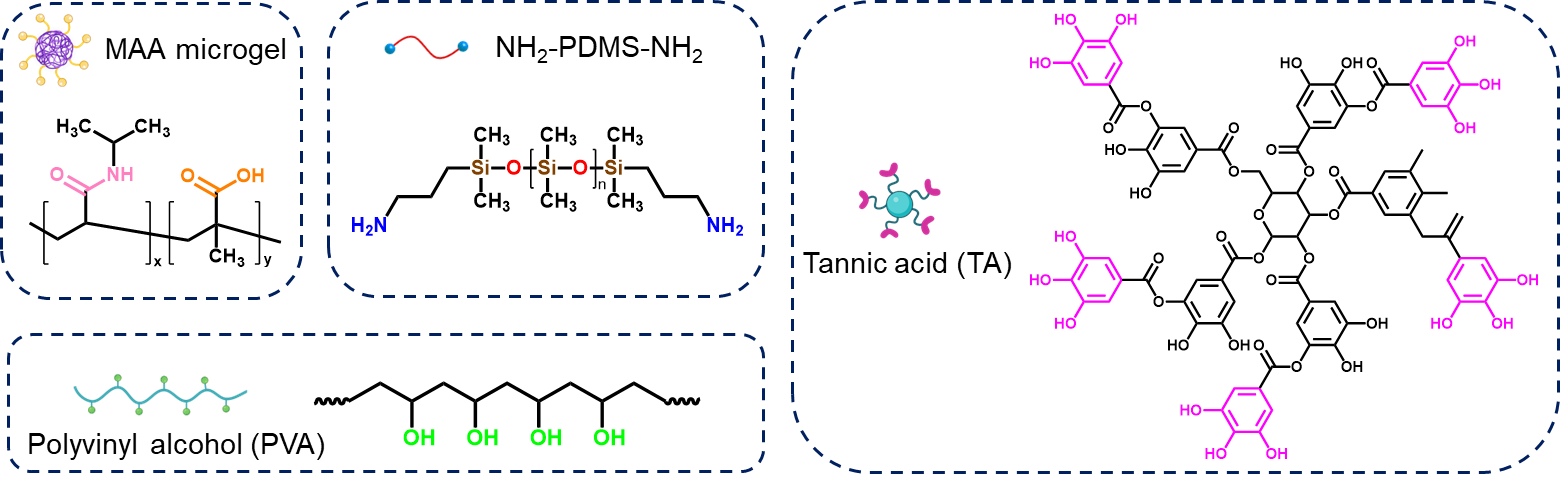


Figure S19. Schematic and corresponding molecular structures of PNIPAM*-co-*MAA, NH_2_-PDMS-NH_2_, PVA, and TA.


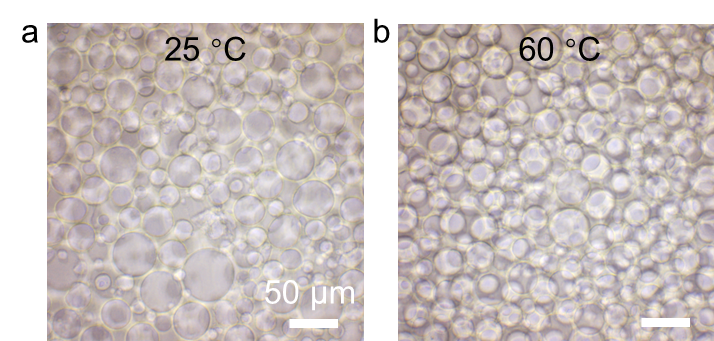


Figure S20. The morphology of octane-in-DMF Pickering emulsion droplets at (a) 25 °C and (b) 60 °C.

Figure S21. Angular frequency sweep for oscillatory shear complex viscosity for a non-covalent octane-in-DMF PO at room temperature.


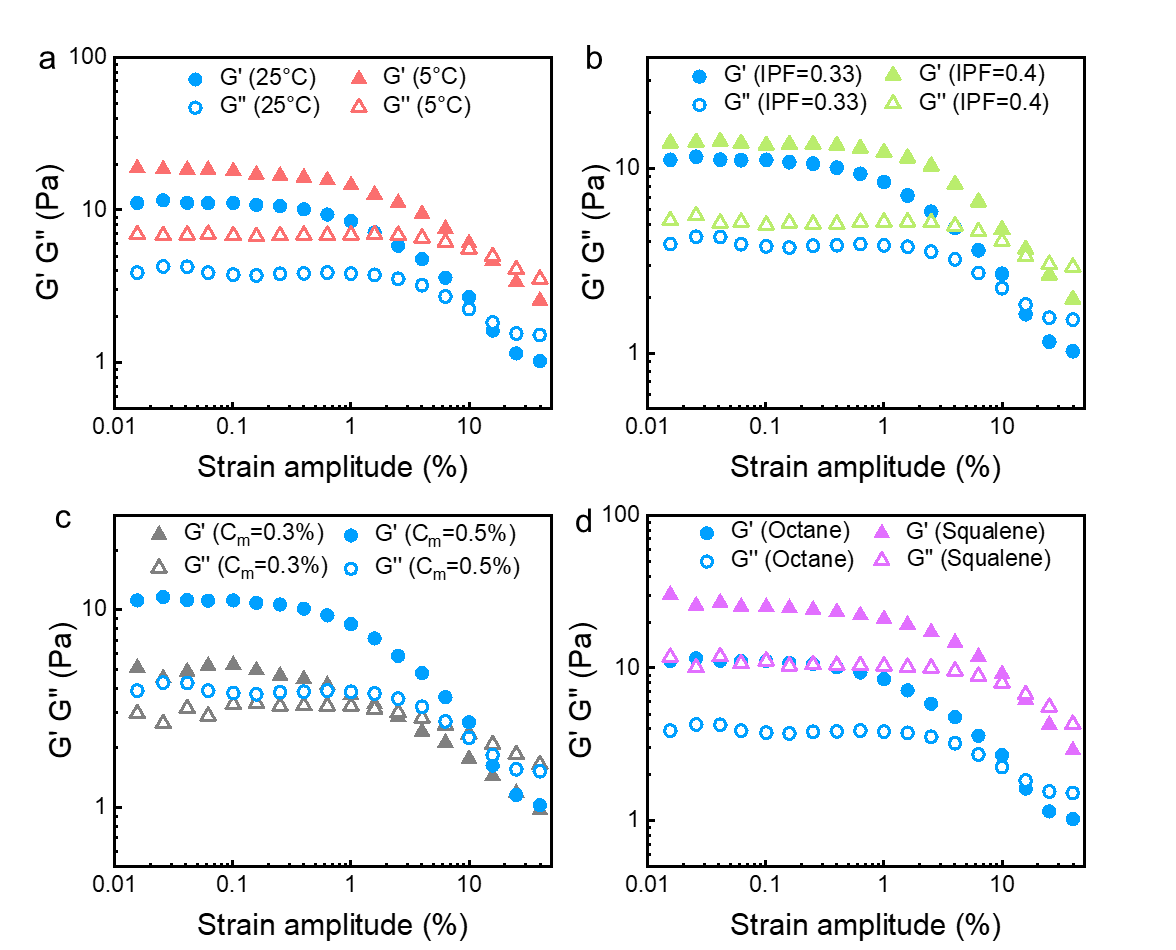


Figure S22. Elasticity and viscosity of non-covalent POs with different emulsification parameters including different (a) temperatures, (b) internal phase fractions, (c) microgel concentrations, and (d) non-polar oil phase.


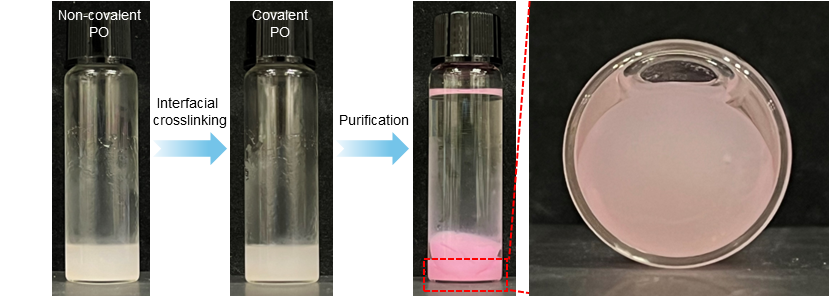


Figure S23. Optical images showing the interfacial crosslinking and purification of the covalent PO templated from the non-covalent PO.


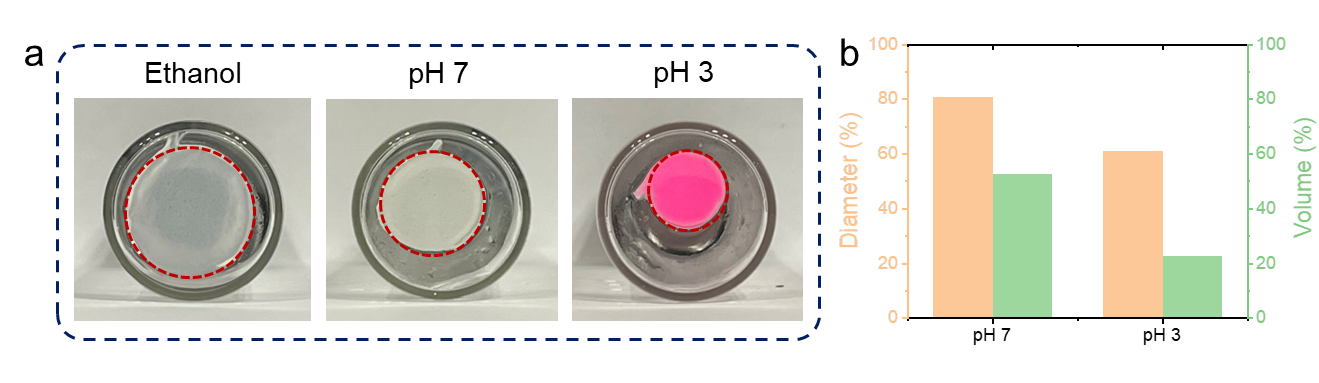


Figure S24. (a) Optical images showing the morphology of the covalent PO in different media. (b) Size and volume variation of the covalent PO in aqueous solutions at pH 7 and pH 3 compared to that in ethanol.


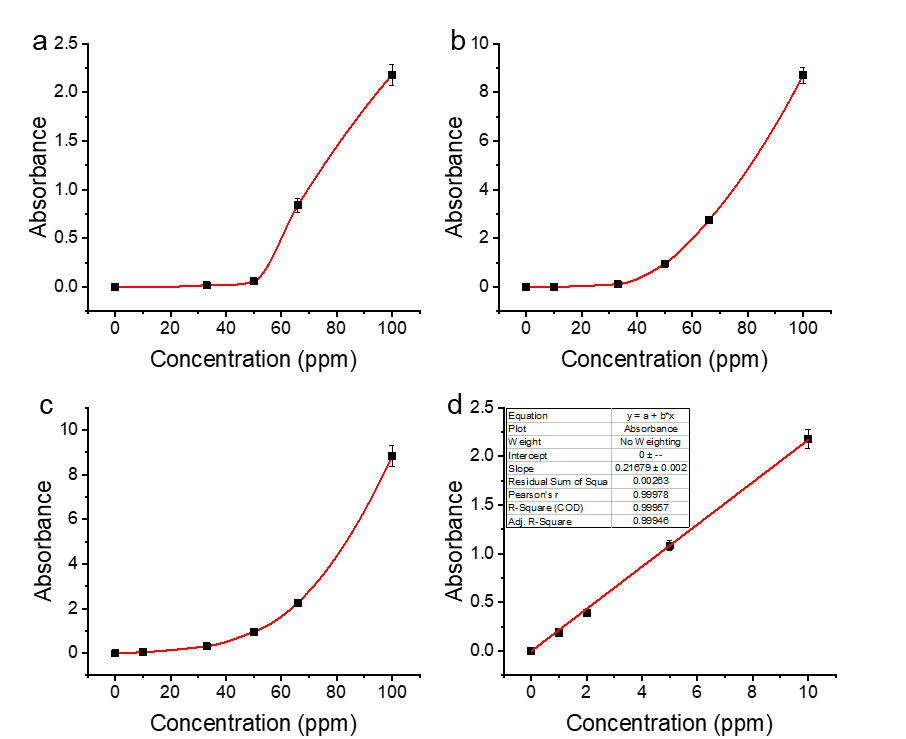


Figure S25. The calibration curves for RB molecules in (a) DMF, (b) DMF containing 1 vol% HA, (c) DMF containing 3 vol% HA, and (d) water at pH 3 based on UV-vis absorption spectra.


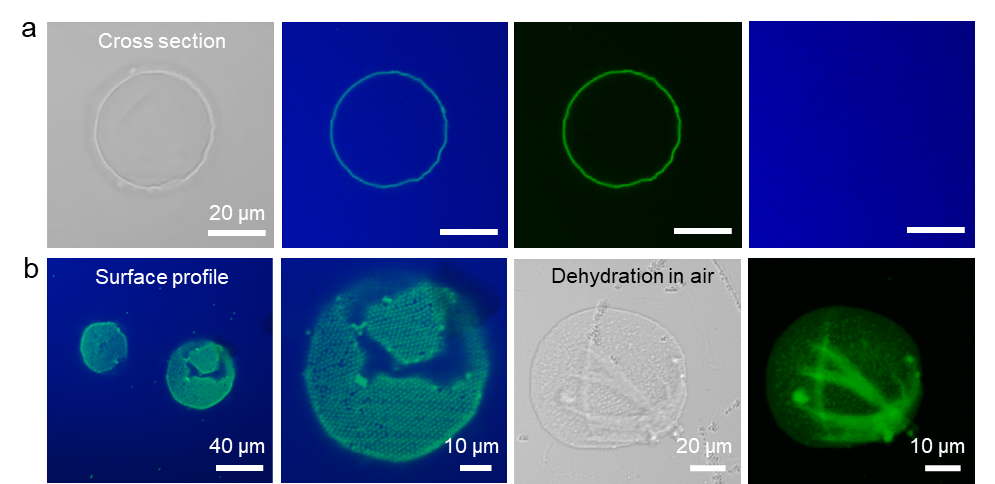


Figure S26. Optical microscopy images and CLSM images of the (a) cross section and (b) surface profile of covalent microgelsomes dispersed in ethanol. Perylene was added in the solution as an indicator.

# References

[1] H. J. Berendsen, D. van der Spoel, R. van Drunen, *Computer physics communications* **1995**, *91*, 43-56.

[2] K. Vanommeslaeghe, E. Hatcher, C. Acharya, S. Kundu, S. Zhong, J. Shim, E. Darian, O. Guvench, P. Lopes, I. Vorobyov, *Journal of computational chemistry* **2010**, *31*, 671-690.

[3] T. Darden, D. York, L. Pedersen, *The Journal of chemical physics* **1993**, *98*, 10089-10092.

[4] S. Jo, T. Kim, V. G. Iyer, W. Im, *Journal of computational chemistry* **2008**, *29*, 1859-1865.

[5] L. Martínez, R. Andrade, E. G. Birgin, J. M. Martínez, *Journal of computational chemistry* **2009**, *30*, 2157-2164.
